# The mechanism of cell death induced by silver nanoparticles is distinct from silver cations

**DOI:** 10.1186/s12989-021-00430-1

**Published:** 2021-10-14

**Authors:** Monica M. Rohde, Christina M. Snyder, John Sloop, Shane R. Solst, George L. Donati, Douglas R. Spitz, Cristina M. Furdui, Ravi Singh

**Affiliations:** 1 Department of Cancer Biology, Wake Forest School of Medicine, Medical Center Blvd., Winston-Salem, NC 27157 USA; 2 Department of Chemistry, Wake Forest University, Winston-Salem, NC 27109 USA; 3 Free Radical and Radiation Biology Program, Department of Radiation Oncology, Holden Comprehensive Cancer Center, University of Iowa, Iowa City, IA 52242 USA; 4 Department of Internal Medicine, Section of Molecular Medicine, Wake Forest School of Medicine, Winston-Salem, NC 27157 USA; 5 Comprehensive Cancer Center of Wake Forest Baptist Medical Center, Winston Salem, NC 27157 USA

**Keywords:** Nanotoxicology, Oxidative stress, Lipid peroxidation, Biosafety, Breast cancer

## Abstract

**Background:**

Precisely how silver nanoparticles (AgNPs) kill mammalian cells still is not fully understood. It is not clear if AgNP-induced damage differs from silver cation (Ag^+^), nor is it known how AgNP damage is transmitted from cell membranes, including endosomes, to other organelles. Cells can differ in relative sensitivity to AgNPs or Ag^+^, which adds another layer of complexity to identifying specific mechanisms of action. Therefore, we determined if there were specific effects of AgNPs that differed from Ag^+^ in cells with high or low sensitivity to either toxicant.

**Methods:**

Cells were exposed to intact AgNPs, Ag^+^, or defined mixtures of AgNPs with Ag^+^, and viability was assessed. The level of dissolved Ag^+^ in AgNP suspensions was determined using inductively coupled plasma mass spectrometry. Changes in reactive oxygen species following AgNP or Ag^+^ exposure were quantified, and treatment with catalase, an enzyme that catalyzes the decomposition of H_2_O_2_ to water and oxygen, was used to determine selectively the contribution of H_2_O_2_ to AgNP and Ag^+^ induced cell death. Lipid peroxides, formation of 4-hydroxynonenol protein adducts, protein thiol oxidation, protein aggregation, and activation of the integrated stress response after AgNP or Ag^+^ exposure were quantified. Lastly, cell membrane integrity and indications of apoptosis or necrosis in AgNP and Ag^+^ treated cells were examined by flow cytometry.

**Results:**

We identified AgNPs with negligible Ag^+^ contamination. We found that SUM159 cells, which are a triple-negative breast cancer cell line, were more sensitive to AgNP exposure less sensitive to Ag^+^ compared to iMECs, an immortalized, breast epithelial cell line. This indicates that high sensitivity to AgNPs was not predictive of similar sensitivity to Ag^+^. Exposure to AgNPs increased protein thiol oxidation, misfolded proteins, and activation of the integrated stress response in AgNP sensitive SUM159 cells but not in iMEC cells. In contrast, Ag^+^ cause similar damage in Ag^+^ sensitive iMEC cells but not in SUM159 cells. Both Ag^+^ and AgNP exposure increased H_2_O_2_ levels; however, treatment with catalase rescued cells from Ag^+^ cytotoxicity but not from AgNPs. Instead, our data support a mechanism by which damage from AgNP exposure propagates through cells by generation of lipid peroxides, subsequent lipid peroxide mediated oxidation of proteins, and via generation of 4-hydroxynonenal (4-HNE) protein adducts.

**Conclusions:**

There are distinct differences in the responses of cells to AgNPs and Ag^+^. Specifically, AgNPs drive cell death through lipid peroxidation leading to proteotoxicity and necrotic cell death, whereas Ag^+^ increases H_2_O_2_, which drives oxidative stress and apoptotic cell death. This work identifies a previously unknown mechanism by which AgNPs kill mammalian cells that is not dependent upon the contribution of Ag^+^ released in extracellular media. Understanding precisely which factors drive the toxicity of AgNPs is essential for biomedical applications such as cancer therapy, and of importance to identifying consequences of unintended exposures.

**Supplementary Information:**

The online version contains supplementary material available at 10.1186/s12989-021-00430-1.

## Introduction

Silver nanoparticles (AgNPs) are one of the most commercialized nanomaterials for biomedical and industrial applications, and extensive analysis of their toxicity has been performed in a wide variety of organisms [1, 2]. Despite this, precisely how AgNPs kill mammalian cells still is not known. There continues to be debate over whether cytotoxic effects of AgNPs are due to uptake of intact nanoparticles or due to extracellular release of silver cation (Ag^+^). Understanding precisely which factors drive the toxicity of AgNPs is essential to guide their utility for biomedical applications and to identify consequences of unintended exposures.

A major contributing factor to the lack of a definitive answer in the particle versus cation debate is poor material characterization. In many studies, there is a substantial amount of Ag^+^ remaining in the AgNP suspension due to lack of purification following synthesis [3, 4]. Because of this, multiple studies conclude that Ag^+^ is the dominant toxicant present in mixtures of intact AgNPs and Ag^+^ [5–7]. Release of Ag^+^ also can occur due to dissolution of AgNPs in culture media, and Teeguarden and colleagues determined that extracellular release of Ag^+^ was sufficient to drive cytotoxic responses of murine macrophages to AgNP exposure [8]. It will be necessary to use preparations of AgNPs with negligible contamination with Ag^+^ to disambiguate fully the contributions to cytotoxicity of intact AgNPs and dissolved Ag^+^.

A second issue that must be addressed to obtain a definitive answer to the AgNP versus Ag^+^ debate is the heterogeneity of the inherent sensitivity of various types of cells to AgNP suspensions. For example, previous studies show that some breast cancer cell lines are more sensitive to AgNP exposure than non-neoplastic mammary epithelial cells [9–11]. Similarly, there is substantial variability in the sensitivity of ovarian cancer [12], lung cancer [13, 14], and leukemia cell lines [15] to AgNP exposure. Our previous recent results show that sensitivity to Ag^+^ exposure does not correlate with sensitivity to intact AgNPs [11]. This indicates that AgNPs and Ag^+^ may elicit different mechanistic responses in cells with high sensitivity compared to those with low sensitivity.

Ag^+^ can be taken up by cells and enter the cytosol through copper ion transporters [16, 17], but AgNPs are too large to pass through ion channels, and instead are taken up by phagocytic and endocytic pathways [18]. After uptake, AgNPs appear to remain confined to membrane bound vesicles including early and late endosomes, and autophagosomes [11]. Despite this, AgNP-induced damage is observed in almost every part of the cell, including mitochondria [13, 14], nucleus [9], and endoplasmic reticulum [10, 11]. A Trojan Horse mechanism has been proposed whereby AgNPs act as a vehicle that carries silver across the cell membrane followed by intracellular dissolution of AgNPs to release Ag^+^ [19, 20], resulting in ROS production, DNA damage, proteotoxic stress, and apoptosis [21, 22]. Intracellular nanoparticle dissolution likely occurs, but if intracellular Ag^+^ release were the dominant mechanism of AgNP toxicity, then intracellular AgNP mass and surface area would be expected to correlate with sensitivity to AgNPs. This is not always the case [8, 11], indicating other factors may drive sensitivity to AgNPs.

Most studies agree that generation of reactive oxygen species (ROS) and induction of oxidative stress drive Ag^+^ cytotoxicity [23–26]. AgNPs also increase ROS [13] and cause oxidative damage to proteins [9, 11, 13], but there is conflicting evidence regarding the dependency of AgNP-induced cytotoxicity on ROS [15, 27, 28]. Exposure to AgNPs also may increase lipid peroxides in earthworms [29], fish [30], and liver cancer cells [31]. Extensive oxidation of lipids disrupts the physical properties of cell membranes, can cause covalent modification of proteins, and can damage mitochondria and nucleic acids [32]. Lipid peroxides and H_2_O_2_ are capable of spreading damage throughout cells, but their relative importance in the toxicity of AgNPs and Ag^+^ has not been studied.

It is not clear if AgNP-induced damage differs from that induced by Ag^+^, nor is it known how AgNP or Ag^+^ damage is transmitted from cell membranes, including endosomes, to other organelles. Therefore, the objectives of this work are: first, to determined if there are specific effects of AgNPs that differed from Ag^+^ in cells with high or low sensitivity to either toxicant; second, to determine if AgNPs and Ag^+^ cause similar or different forms of oxidative damage, cell stress, and cell death; and third, to determine if H_2_O_2_ or lipid peroxide generation differs between AgNPs and Ag^+^, and to assess their roles in transmission of damage.

## Material and methods

### Silver nanoparticles

25 nm in diameter spherical AgNPs stabilized with polyvinylpyrrolidone (PVP) were purchased from nanoComposix, Inc (San Diego, CA, USA). The manufacturer specified a mass ratio of 17% Ag to 83% PVP for the nanoparticles. Nanoparticles were dispersed at a concentration of 5 mg/mL (Ag mass, excluding PVP) in phosphate buffered saline, pH 7.4, without calcium or magnesium (PBS) (Invitrogen, Carlsbad, CA, USA) by bath sonication for 30 min, and then were diluted in cell culture medium to the final concentration listed in the figures prior to addition to the wells containing cells.

### Cell culture

SUM159 cells were purchased from Astrerand (now BioIVT, Westbury, NY, USA). iMEC cells were provided by Dr. Elizabeth Alli in the Department of Cancer Biology at Wake Forest School of Medicine. SUM159 cells expressing doxycycline inducible catalase (SUM159^cat^) were generated as follows. Briefly, the doxycycline-inducible catalase overexpression plasmid was generated as previously described [33]. Lentivirus was produced in the TSA201 cell line using pCMV-VSV-G and psPAX2 helper vectors (Addgene, Caimbridge, MA, USA). Sum159 cells were plated and allowed to grow for 48 h, and then virus was added to cells with 8 µg/mL of polybrene every 24 h for two days. After transduction, cells were selected with 3 µg/mL puromycin. Surviving cells were plated in 100-mm dishes with 100 cells per dish. Clones were grown for 8 days, and then several colonies were picked and expanded. To test for maximal catalase overexpression, cells were treated with 2 µg/mL of doxycycline for 48 h. Protein concentration was determined using the Lowry Assay. Increased catalase activity was verified by measuring the decomposition of H_2_O_2_ by cell lysates as previously described [34]. The clone with the greatest catalase activity following induction was selected for use in the current studies. Low passage stocks of cells were stored in liquid nitrogen and maintained by the Wake Forest Comprehensive Cancer Center Cell Engineering Shared Resource. iMEC cells were grown in DMEM/F12 basal media (Lonza, Morristown, NJ, USA) supplemented with 10 µg/ml insulin, 20 ng/ml hEGF, 0.5 μg/ml hydrocortisone (all from Sigma-Aldrich), and 10% fetal bovine serum (FBS) (Gibco; ThermoFisher Scientific, Waltham, MA, USA). SUM159 and SUM159^cat^ cells were grown in Hams F12 basal media (Lonza) supplemented with 5 µg/ml insulin, 10 mM HEPES, and 1 μg/ml hydrocortisone (all from Sigma-Aldrich), and 5% FBS (Gibco). SUM159^cat^ cells were treated with 2 µg/mL of doxycycline 24 h before subsequent treatments to induce catalase expression and were maintained in doxycycline for the duration of each experiment. Cell monolayers were grown in tissue culture treated plastics purchased from Corning Life Sciences (Corning, NY, USA) or on glass coverslips (Warner Instruments Corporation, Hamden, CT, USA). Cells were provided fresh media and passaged twice weekly, and were maintained in culture for no longer than 3 months before new cultures were established from low passage frozen stocks. All cells were verified to be free from mycoplasma contamination by periodic testing using the MycoAlert Mycoplasma Detection Kit (Lonza, Morristown, NJ, USA).

### Nanoparticle characterization

The hydrodynamic diameter of AgNPs in PBS or SUM159 cell culture media was determined using a Nanosight NS500 (Malvern Instruments, Malvern, UK) running NTA 3.2 Dev Build 3.2.16 software. Data were acquired by analyzing a 60 s video taken by the instrument with camera level 6 and a detection threshold of 7. Five measurements were obtained per sample. A Zetasizer Nano ZS90 (Malvern Instruments) running software version 7.12 was used for ζ-potential measurements. Triplicate measurements were made for each sample.

### MTT assay

Cells were seeded on 96 well plates at a density of 3500–5000 cells per well (depending upon cell line) in 100 µL of complete media, recovered for 24 h, and then were exposed to AgNPs or AgNO_3_ (Sigma-Aldrich) as a source of Ag^+^ in 100 µL of complete media containing the indicated concentrations of silver listed in the figures. After 72 h, media containing AgNPs or Ag^+^ were replaced with 200 µL of media containing 0.5 mg/mL 3-(4,5-dimethylthiazol-2-yl)-2,5-diphenyltetrazolium bromide (MTT; Sigma-Aldrich) and incubated for 2 h at 37 C. Media was removed, and cells were lysed in 200 uL of DMSO and absorbance read using a Molecular Devices (San Jose, CA, USA) Emax Precision Microplate Reader at 595 nm. To control for contributions to absorbance due to AgNPs, the absorbance of wells treated with AgNPs but which do not receive MTT also was measured. Comparisons of curve fitting and statistical analysis of background corrected and raw absorbance measurements fell within the standard deviation of normal sample variation, and therefore any contribution of AgNP absorbance was deemed negligible to the overall results.

### Western blots

Cells were plated on 6 cm tissue culture dishes (Corning Life Sciences) at a density of 1 × 10^6^ cells in 4 mL of complete medium. Cells were allowed to recover for 48 h and then were exposed to AgNPs or Ag^+^ for 24 h at 37 °C. Medium was removed and cells were washed twice with ice cold PBS before lysates were collected using Mammalian Protein Extraction Reagent (Thermo Fisher Scientific) supplemented with 1% Halt Protease & Phosphatase Inhibitor Cocktail (Thermo Fisher Scientific). Protein concentration was determined for each sample using a Pierce bicinchoninic acid (BCA) protein assay kit (Thermo Fisher Scientific) according to manufacturer’s instructions. Proteins were size fractionated by gel electrophoresis and then transferred to a nitrocellulose; Thermo Fisher Scientific) membrane. Nonspecific binding was blocked by incubation for 30 min at room temperature with tris-buffered saline containing 5% bovine serum albumin (Sigma) or 5% blotting-grade blocker (Bio-Rad). Membranes were blotted overnight at 4 °C with 1:1000 dilutions of primary antibodies phospho-eIF2α (9721), eIF2α (9722), phospho-JNK (9255), JNK (9252), GAPDH (2118), β-actin (4970) purchased from Cell Signaling Technologies (Danvers, MA, USA), or 4-HNE (MAB3249-SP) purchased from Thermo Fisher Scientific, washed, and then incubated with anti-rabbit (Cell Signaling Technologies) or anti-mouse (Cell Signaling Technologies) horseradish peroxidase (HRP)-conjugated secondary antibodies; (diluted 1:1000) for 1 h at room temperature. Immunoreactive products were visualized by chemiluminescence (SuperSignal West Pico Plus, Thermo Fisher Scientific). After visualization, blots were stripped of antibody binding by incubating with Restore Plus western blot stripping buffer (Thermo Fisher Scientific) for 5 min, before blocking and re-probing with additional primary antibodies.

### Protein aggregation assays

Cells were plated on 18 mm coverslips in a 12-well plate (Warner Instruments Corporation, Hamden, CT, USA) at a density of 250,000 cells in 1 mL of complete medium. Cells were allowed to recover for 72 h and then were exposed to AgNPs or Ag^+^ for 24 h at 37 C. Medium was removed and cells were fixed with 4% formaldehyde solution and permeabilized (0.5% Triton X-100, 3 mm EDTA, pH 8.0). Cells were then stained with Proteostat Aggresome Detection Reagent (1:1000) and Hoechst 33,342 (1:1000) (Enzo Biosciences, Ann Arbor, MI) diluted in 1X PBS for 30 min, washed twice with PBS, and coverslips were mounted on glass slides with Prolong Gold antifade reagent (Invitrogen). Fluorescence was visualized using an Olympus FV1200 spectral laser scanning confocal microscope.

### Lipid peroxidation assays

Cells were plated on 18 mm coverslips in a 12-well plate (Warner Instruments Corporation, Hamden, CT, USA) at a density of 250,000 cells in 1 mL of complete medium for microscopy experiments and on 6-well plates at a density of 500,000 cells per well in 2 mL of complete medium for flow cytometry experiments. Cells were allowed to recover for 24 h and then were exposed to AgNPs or Ag^+^ for 24 h at 37 °C. Medium was removed and fresh media containing 10 µM of the lipid peroxide specific dye, Liperfluo (Dojindo Molecular Technologies, Rockville, MD,) was added for 30 min. Cells were then washed twice with PBS and fluorescence was measured using an Olympus FV1200 spectral laser scanning confocal microscope and a FACS Canto II Analyzer (BD Biosciences). Analysis of the data was performed using FCS express version 7 (De Novo Software, Glendale, CA, USA).

### Protein oxidation assays

Cells were plated on 6-well plates at a density of 500,000 cells per well in 2 mL of complete medium. Cells were allowed to recover for 24 h and then were exposed to AgNPs or Ag^+^ for 24 h at 37 C. Medium was removed and fresh media containing 50 µM of DCP-NEt_2_-Coumarin (DCP-NEt_2_C) prepared as previously described [35] was added for 30 min. Cells were then washed twice with PBS, fixed with 100% methanol and fluorescence was measured using a FACS Canto II Analyzer (BD Biosciences). Analysis of the data was performed using FCS express version 7 (De Novo Software, Glendale, CA, USA). To control for any potential contribution to the fluorescence profile due to AgNPs themselves, unstained AgNP treated and untreated cells were prepared and analyzed as described above. As shown in Additional file 1: Fig. S1A, the fluorescence profiles overlapped, indicating no difference between the two groups, and the magnitude of fluorescence was less than 0.5% of the DCP-NEt_2_C stained, untreated controls. Because these background measurements fell within the standard deviation of normal sample variation, any contribution of AgNP absorbance was deemed negligible to the overall results.

### Clonogenic assay

Cells were grown to log phase in their respective media, trypsinized, washed in PBS, and plated on six-well plates at a density of 300 cells per well and were allowed to adhere for 24 h. Increasing concentrations of AgNPs were added to each well and incubated for 24 h at 37 °C. For each condition, 6 wells were used. Cells were incubated with AgNPs with or without PEG-catalase (Sigma-Aldrich) (100 U/mL) for 24 h, and then culture media was removed. The cells were washed with PBS, and fresh media was added and replaced every 2–3 days. Fourteen days after plating, the cells were washed and fixed with methanol, glacial acetic acid, and water (1:1:8 [vol:vol:vol]), then stained with crystal violet. Colonies of at least 50 cells were counted by hand. All data are expressed as plating efficiency relative to the relevant control in the absence of AgNPs.

### Transmission electron microscopy

SUM159 or iMEC cells were grown in 6-well tissue culture dishes. Cells were treated with AgNPs (150 µg/mL) for 1 h and were washed thoroughly with PBS to remove AgNPs not bound or internalized by cells. Fresh cell culture media was added and cells were incubated for 5 h more before fixation in 2.5% gluteraldehyde at 4 °C overnight. Next, cells were scraped from the wells, pelleted, embedded in resin, cut into ultrathin sections (80 nm), and placed on copper coated formvar grids by the Wake Forest Comprehensive Cancer Center Cellular Imaging Shared Resource. Cells were imaged without additional staining to facilitate the detection of AgNPs using a Tecnai Spirit transmission electron microscope (FEI Company; Hillsboro, OR, USA).

### ROS detection

SUM159 and iMEC cells were plated on 8-well coverslip-bottom chamber slides (EMD Millipore, Burlington, MA, USA) at a density of 30,000 cells per well in 400 µL of complete medium. Cells were allowed to recover for 48 h and then were exposed to AgNPs or Ag^+^ with or without PEG-Catalase (100 units/mL) for 24 h at 37 °C. Medium was removed and cells were washed with PBS, and incubated with 10 μM 2’,7’-dichlorodihydrofluorescein diacetate (H_2_DCF-DA) (Invitrogen) or PeroxyOrange-1 (PO1) (Tocris) for 30 min at 37 °C. Fluorescence was visualized using an Olympus FV1200 spectral laser scanning confocal microscope.

### ICP-MS

A freshly prepared suspension of AgNPs dispersed in PBS at 5 mg/mL was transferred to a 5000 MWCO VivaSpin column (Viva products, Littleton, MA) and centrifuged at 3000 × g for 15 min. The same suspension of AgNPs was stored at 4 °C for 1 week and processed as described. The flow through, containing any soluble silver, was collected and stored at 4 °C. Triplicate, 50 µL samples were then digested with 1 mL of concentrated HNO_3_, 2 mL of 30% H_2_O_2_ and 7 mL of distilled-deionized water using a microwave-assisted digestion system (Ethos UP, Milestone, Sorisole, Italy). The digested samples were diluted to a final acid concentration of 2% v/v before Ag determination by inductively coupled plasma mass spectrometry (ICP-MS). Trace metal grade HNO_3_ (Fisher, Pittsburgh, PA, USA), and distilled-deionized water (18 MΩ cm, Milli-Q®, Millipore) were used to digest samples and prepare all solutions. Low trace metals hydrogen peroxide (Veritas, Columbus, OH, USA) was also used for sample digestion. Standard reference solutions used for calibration were prepared in 2% v/v HNO_3_ from a 10 mg/L Ag stock (High-Purity Standards, Charleston, SC, USA). A tandem ICP-MS (8800 ICP-MS, Agilent Technologies, Tokyo, Japan), equipped with a SPS 4 automatic sampler, a Scott-type double pass spray chamber operated at 2 °C, and a glass concentric nebulizer were used in all determinations. Helium gas (> = 99.999% purity, Airgas) was used in the ICP-MS’s collision/reaction cell to minimize potential spectral interferences, while monitoring the ^109^Ag isotope in single quadrupole mode. Other relevant instrument operating conditions such as radio frequency applied power, sampling depth, carrier gas flow rate, and reaction gas flow rate were 1550 W, 10.0 mm, 1.05 L/min, and 4.0 mL/min, respectively.

### Apoptosis/necrosis analysis

SUM159 and iMEC cells were plated at a density of 500,000 cells per well in a 6 well plate and allowed to adhere overnight. Cells were then treated with AgNPs, Ag^+^ or vehicle. After 24 h cells were washed twice with PBS, trypsinized, and resuspended in their respective media. Cells were then pelleted by centrifugation at 320 × g for 5 min. Cells were washed with PBS and pelleted again by centrifugation at 320 × g for 5 min. FITC-conjugated Annexin V and ethidium homodimer III staining was performed per the manufacturer’s instructions (Biotium, Fremont, CA). Labeled cells were analyzed on a FACS Canto II Analyzer (BD Biosciences). Analysis of the data was performed using FCS express version 7 (De Novo Software, Glendale, CA, USA). Unstained samples were included to control for any potential interference of AgNPs with flow cytometry. There was no detectable change in background fluorescence in the unstained samples, indicating that AgNPs did not interfere with the assay [Additional file 1: Fig. S1B].

### Statistical analysis

Analysis was performed using GraphPad Prism 9.0 software as described in the figure legends. The number of experimental and biological replicates used for each experiment is included in the figure legends.

## Results

### Contamination of AgNP suspensions with as little as 1% Ag^+^ by mass can mask AgNP-specific cytotoxicity

**Fig. 1:**
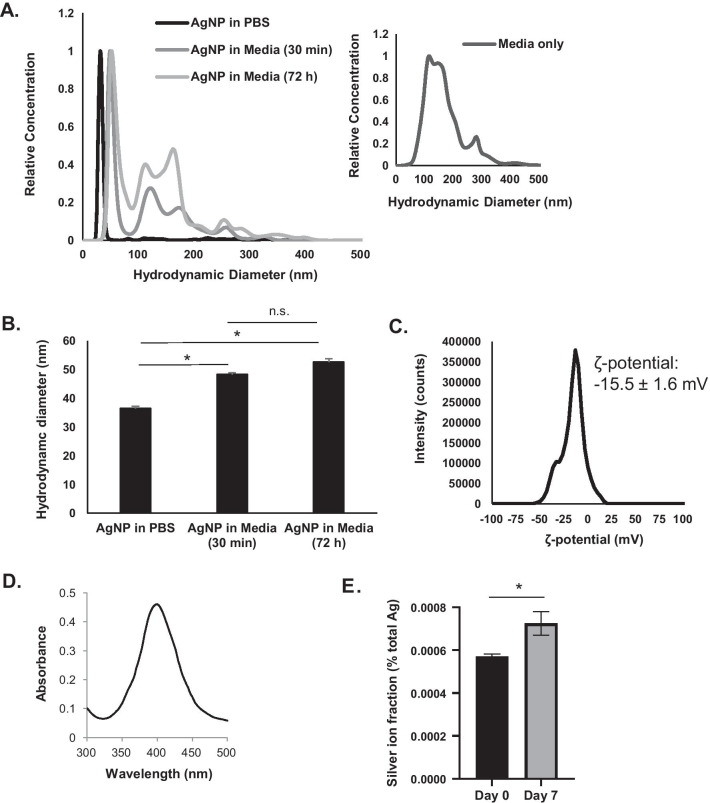
No evidence of AgNP aggregation and minimal release of Ag^+^ were detected following storage of AgNPs. **A** Nanoparticle tracking analysis was used to determine the hydrodynamic diameter of AgNPs in PBS, 30 min after dilution in cell culture media, and 72 h after dilution in cell culture media. The inset to the right shows the size distribution of particulate material in the media itself. **B** The peak from the hydrodynamic diameter for AgNPs measured in (**A**) was identified and the median values of five measurements per condition ± standard deviation are shown. **C** The ζ-potential distribution of AgNPs dispersed PBS at pH 7.4 after 100X dilution in water is shown. **D** The UV/visible light absorbance spectrum of AgNPs (arbitrary units (a.u.) is shown. **E** The fraction of Ag^+^ relative to total silver mass in a freshly prepared dispersion of AgNPs or following storage for 7 days is show. Statistical analysis was performed by Student T-test. Significant differences are indicated (*p < 0.05); n.s. not significant

We purchased 25 nm, PVP coated, AgNPs from nanoComposix Inc. (Lot #ALJ0052-MGM2463A) and stored them as a lyophilized powder in the dark at 4 °C. Prior to use in cell culture experiments, we characterized the hydrodynamic diameter of AgNPs after hydration in PBS or dilution in cell culture media using nanoparticle tracking analysis (NTA) (Fig. 1A, B). A single sharp peak corresponding to AgNPs was observed for size ranges under 100 nm. Additional peaks in the size range greater than 100 nm were attributable to the media, possibly indicating the presence of exosomes or protein aggregates. The hydrodynamic diameter measured at the AgNP peak increased from 36.5 ± 0.7 nm in PBS to 48.3 ± 0.6 after incubation in cell culture media for 30 min, likely due to formation of a protein corona. After 72 h in media, a non-significant (*p* > 0.05) size increased to 52.6 ± 1.2 was detected and there was no evidence of large aggregate formation or sedimentation. The ζ-potential in PBS at pH 7.4 was -15.5 ± 1.6 mV (Fig. 1C), and the plasmon resonance peak was 402 nm, which is typical for 25 nm, spherical AgNPs (Fig. 1D). We quantified the amount of soluble silver (Ag^+^) present in the AgNP suspension by separating the solid and soluble fractions using a size exclusion, spin column. The silver content of the filtered solution was quantified by ICP-MS and the mass of silver in the filtrate was compared to the total mass of silver in the initial suspension of AgNPs. As shown in (Fig. 1D), a freshly prepared suspension of AgNPs contained only 0.000571% Ag^+^ by mass. Following 7 days of storage, there was a slight increase in the Ag^+^ fraction to 0.000725%.

**Fig. 2:**
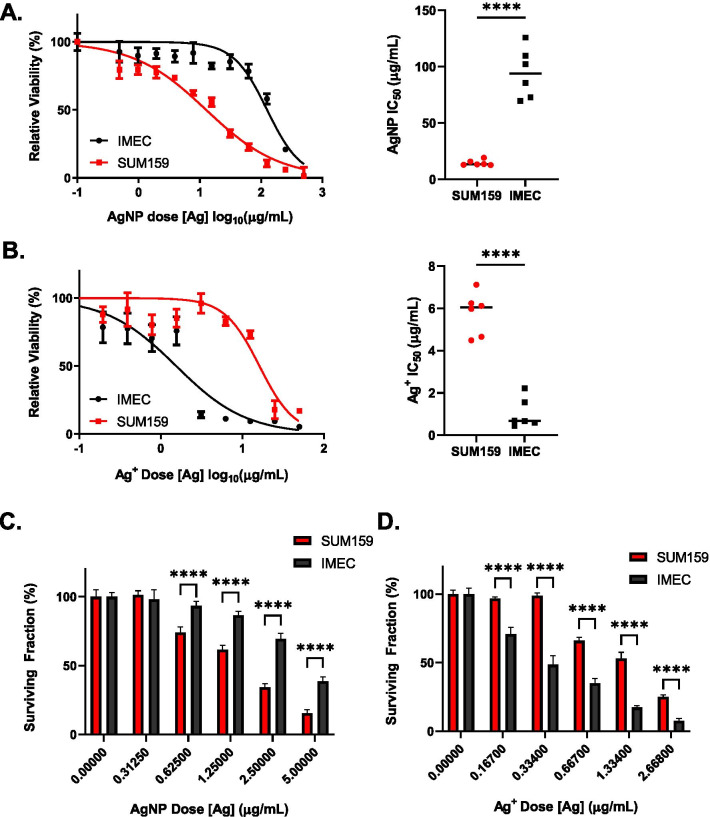
Sensitivity to AgNPs is independent from sensitivity to silver ion. Representative dose response curves and IC_50_ following 72 h **A** AgNP or **B** Ag^+^ exposure. Cell viability following 72 h AgNP or Ag^+^ exposure was quantified by MTT assay and IC50 was determined using GraphPad Prism. Data used to calculate IC_50_s were obtained from 6 technical replicates per dose and 3 independent experiments (biological replicates). Statistical analysis was performed by two-was ANOVA and post-hoc Tukey Test. Significant differences are indicated (****p < 0.0001). Long-term proliferative potential was assessed via clonogenic assay following 24 h **C** AgNP or **D** Ag^+^ exposure. The data are presented as relative surviving fraction based upon clonogenic growth normalized to the plating efficiency. Statistical analysis was performed by two-way ANOVA followed by post-hoc Tukey Test. Statistical differences are indicated (****p < 0.0001)

Next, we asked if this fraction of Ag^+^ was sufficient to drive cytotoxic responses to AgNPs in cells with high or low sensitivity to AgNPs. Previously, we found that AgNPs were lethal to a subset of breast cancer cell lines, including SUM159 cells, at doses that had little effect on the viability of various models of normal breast epithelia, including iMEC cells [11]. Therefore, we used SUM159 cells as a model for an AgNP sensitive cell line, and iMEC cells as a model for an AgNP insensitive cell line. Both cell types were exposed to increasing doses of AgNPs or Ag^+^ for 72 h. Because aging of AgNPs or Ag^+^ in cell culture media can reduce their cytotoxicity [8], we minimized any effects due to interactions with media components by using dilutions of AgNPs and Ag^+^ in cell culture media within 30 min of preparation. Viability was assessed by MTT assay, and the half-maximal inhibitory concentration (IC_50_) of AgNPs or Ag^+^ in relation to viability was calculated. Based upon differences in IC_50_, we observed SUM159 cells were approximately 6.5-fold more sensitive to AgNP exposure compared to iMEC cells (IC_50_ of 15.1 vs 97.9 µg/mL respectively) (Fig. 2A). In contrast, SUM159 cells were approximately 5.6-fold less sensitive to Ag^+^ compared to iMEC cells (IC_50_ of 1.03 vs 5.8 µg/mL respectively) (Fig. 2B). We verified these differences in sensitivity to AgNPs and Ag^+^ by evaluating clonogenic growth after AgNP or Ag^+^ exposure. In agreement with the results from the MTT assay, clonogenic assays showed that AgNPs were significantly more cytotoxic towards SUM159 cells compared to iMEC cells (Fig. 2C), while Ag^+^ treatment was significantly more cytotoxic towards iMEC cells compared to SUM159 cells (Fig. 2D). Based upon the fraction of Ag^+^ present in the AgNP suspension as determined by ICP-MS (Fig. 1D), a dose of 100 µg/ml of intact AgNPs at most would contain 0.000725 µg/ml of Ag^+^. This concentration of Ag^+^ did not decrease the viability of either cell line, indicating that the cytotoxicity induced by AgNPs was not due to the small amount of Ag^+^ remaining in the suspension. Because SUM159 cells were significantly more sensitive to AgNPs compared to iMEC cells but were less sensitive to Ag^+^, these data suggested the cytotoxicity induced by intact AgNPs was distinct from that of Ag^+^.

**Fig. 3:**
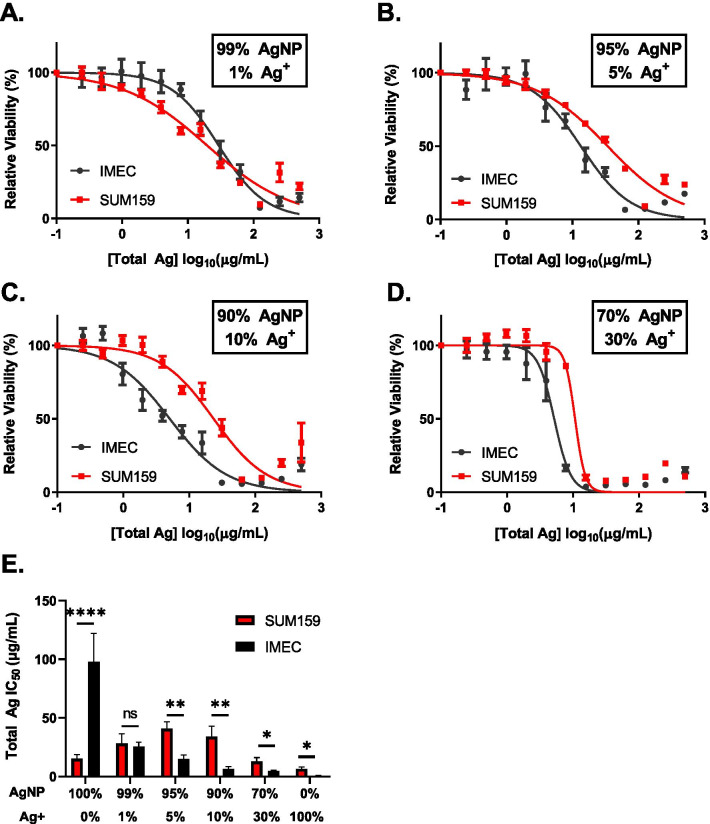
Presence of Ag^+^ in AgNP dispersions masks differences in sensitivity of cell lines to AgNPs. Representative dose response curves following 72 h AgNP-Ag^+^ exposure in the following proportions **A** 99% AgNP/1% Ag^+^, **B** 95% AgNP/5% Ag^+^, **C** 90% AgNP/10% Ag^+^, **D** 70% AgNP/30% Ag^+^. Cell viability following AgNP-Ag^+^ exposure was quantified by MTT assay. **E** IC_50_ was determined using GraphPad Prism. Data used to calculate IC50s were obtained from 6 technical replicates per dose and 2–3 independent experiments. Statistical analysis was performed by two-way ANOVA followed by post-hoc Sidaks test. Significant differences are indicated (*p < 0.05; **p < 0.01; ****p < 0.0001)

To determine what fraction of Ag^+^ present in a suspension of AgNPs could affect the overall cytotoxicity profile, we exposed cells to increasing concentrations of AgNP and Ag^+^ mixtures containing 99–70% intact AgNPs with 1–30% Ag^+^ by total silver mass (Fig. 3A–D). We then calculated the IC_50_ for each mixture based upon total silver concentration (Fig. 3E). As noted above, iMEC cells were relatively insensitive to intact AgNPs, but highly sensitive to Ag^+^. In agreement with this, there was a shift in the IC_50_ in iMEC cells for 100% AgNPs from 97.9 µg/mL (Fig. 2A) to 25.6 µg/mL for a mixture of 99% AgNPs/1% Ag^+^ (Fig. 3A, E). As the percentage of Ag^+^ increased, the IC_50_ in iMEC cells dropped to 15.1 µg/mL at 95% AgNP/5% Ag^+^ (Fig. 3B, E), 6.3 µg/mL at 90% AgNP/10% Ag^+^ (Fig. 3C, E), and 4.8 µg/mL at 70% AgNP/30% Ag^+^ (Fig. 3D, E). The opposite trend was observed for SUM159 cells, which were sensitive to intact AgNPs but comparatively insensitive to Ag^+^ (Fig. 2A, B). There was a small but not statistically significant (*p* > 0.05) increase in IC_50_ of a 99% AgNP/1% Ag^+^ compared to 100% AgNP (28.2 vs 15.1 µg/mL respectively; Figs. 2A and 3A, E). The IC_50_ increased to 40.8 µg/mL for 95% AgNP/5% Ag^+^ (40.78 µg/mL; Fig. 3B, E). The IC_50_ for the 90% AgNP/10% Ag^+^ (33.9 µg/mL; Fig. 3C, E) also was greater than that of the 100% AgNP treatment. Only for the 70% AgNP/30% Ag^+^ treatment of SUM159 cells did the IC_50_ (12.9 µg/mL) fall below that of the 100% AgNP treatment (Fig. 3D, E). As shown in Fig. 3E, as little as a 1% Ag^+^ mass fraction in an AgNP suspension narrowed the difference in sensitivity of iMEC and SUM159 cells compared to exposure to intact AgNPs free from Ag^+^. Mixtures of AgNPs containing 5% or greater mass fractions of Ag^+^ were significantly less cytotoxic to SUM159 than they were to iMEC cells, which is the opposite of what was observed for AgNPs in the absence of Ag^+^. These data show that depending on cell line and relative sensitivity to intact AgNPs versus Ag^+^, the fraction of Ag^+^ present in an AgNP suspension can mask or even reverse any inherent differences between the cytotoxic responses of cells to intact AgNPs.

### Both AgNP and Ag^+^ increase intracellular H_2_O_2_, but only Ag^+^ cytotoxicity is dependent upon H_2_O_2_

**Fig. 4:**
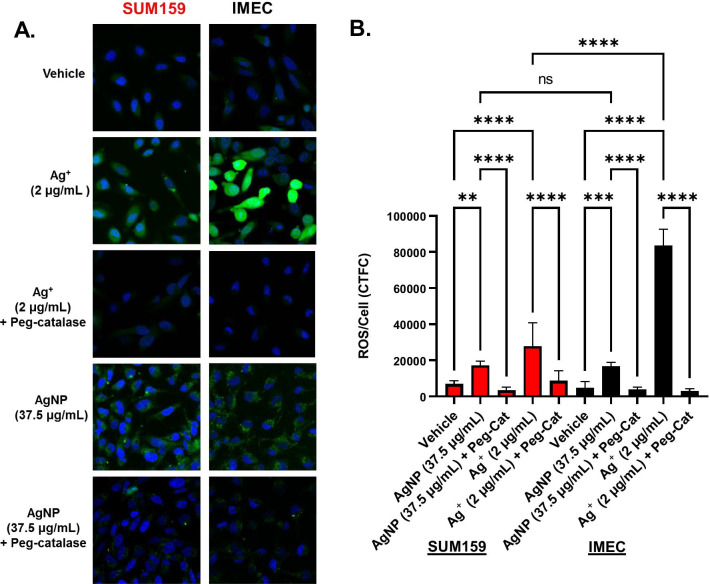
Both AgNPs and Ag^+^ increase ROS. **A** SUM159 and iMEC cells were treated with either Ag^+^ or AgNPs for 24 h with and without 100 IU peg-catalase. Cells were then incubated with PBS containing H_2_DCF-DA for 30 min and fluorescense was measured using confocal microscopy. Images were quantified in (**B**). Results shown in **A** as representative images from 3 independent experiments. Data in **B** is plotted as corrected total cell fluorescences ± SEM of at least 10 cells per image for 3 images. Statistical analysis was performed by one-way ANOVA followed by post-hoc Sidaks test. Significant differences are indicated (**p < 0.01; ***p < 0.001; ****p < 0.0001)

Having established that AgNPs and Ag^+^ exhibit distinct cytotoxic responses, we looked for mechanistic differences that could account for this. First, we examined total ROS in SUM159 and iMEC cells after 24 h exposure to AgNPs or Ag^+^ by quantifying changes in fluorescence intensity of CM-H_2_DCF-DA. Treatment of cells with PEG-catalase, a cell permeable antioxidant that enzymatically catalyzes the reduction of H_2_O_2_ to water and O_2_, was used as a control to determine the contribution of H_2_O_2_ to CM-H_2_DCF-DA fluorescence signal. Unstained samples treated with AgNPs were imaged in parallel to ensure AgNPs did not affect background fluorescence (Additional file 1: Fig. S2). There was a significant (*p* < 0.01) increase in ROS in both cell lines following both Ag^+^ and AgNP treatment, and this increase was blocked by PEG-catalase (Fig. 4A, B). Following exposure to Ag^+^, significantly more ROS was generated by iMEC cells compared to SUM159 cells, which is in agreement with their relative sensitivity to Ag^+^. CM-H_2_DCF-DA fluorescence after Ag^+^ exposure was observed throughout the entire volume of both cell lines. In contrast, similar increases in ROS were observed for AgNP treated iMEC and SUM159 cells, and the CM-H_2_DCF-DA fluorescence after AgNP exposure was punctate and only observed in the cytoplasm. TEM images of AgNPs in iMEC and SUM159 cells show intact nanoparticles in membrane bound vesicles consistent with endosomes (Additional file 1: Fig. S3), indicating that AgNP-induced increases in ROS may be localized to these compartments.

**Fig. 5:**
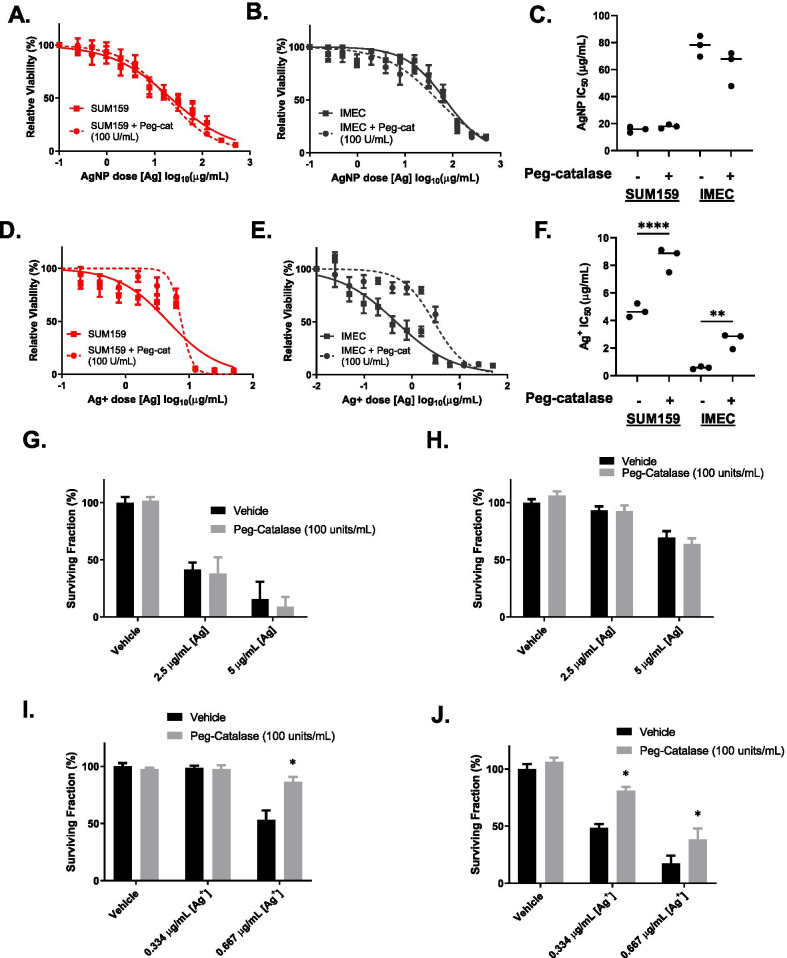
Reduction of hydrogen peroxide through PEG-catalase attenuates Ag^+^ but not AgNP mediated cell death. **A** SUM159 or **B** iMEC cells were exposed to increasing doses of AgNPs with or without 100 units/mL of PEG-catalase. Cell viability following 24 h AgNP exposure was quantified by MTT assay. **C** IC_50_ was calculated using GraphPad Prism. Data used to calculate IC_50_’s were obtained from 6 technical replicates per dose and 3 independent experiments (biological replicates). **D** SUM159 or **E** iMEC cells were exposed to increasing doses of Ag^+^ with or without 100 units/mL of PEG-catalase. Cell viability following 24 h Ag^+^ exposure was quantified by MTT assay. **F** IC_50_ was calculated using GraphPad Prism. Data used to calculate IC_50_’s were obtained from 6 technical replicates per dose and 3 independent experiments (biological replicates). Statistical analysis in **C** and **F** was performed by students T-Test. Statistical differences are indicated (**p < 0.01; ****p < 0.0001). **G**, **H** Long term proliferative potential was assessed via clonogenic assay following 24 h AgNP exposure in the presence of 100 units/mL of PEG-catalase in **G** SUM159 and **H** iMEC cells. **I**, **J** Long-term proliferative potential was assessed via clonogenic assay following 24 h Ag^+^ exposure in the presence of 100 units/mL of peg-catalase in **I** SUM159 and **J** iMEC cells. Data in **G–J** are presented as relative surviving fraction based upon clonogenic growth normalized to plating efficiency. Statistical analysis was performed by one-way ANOVA followed by post-hoc Tukey Test. Statistical differences are indicated (*p < 0.05)

Of the major types of ROS, H_2_O_2_ has the longest biological half-life and is capable of diffusing through cell compartments via aquaporins present in membranes [36]. H_2_O_2_ is known to be a major contributor to the toxicity of Ag^+^ [23, 26] and other redox active metals like iron [37]. We therefore focused on the contribution of H_2_O_2_ to AgNP and Ag^+^ cytotoxicity. PeroxyOrange-1 (PO1) is a specific probe for H_2_O_2_ [38]. Quantification of PO1 staining in AgNP and Ag^+^ treated iMEC or SUM159 cells produced similar results to those observed for CM-H2DCF-DA (Additional file 1: Fig. S4). Because AgNP and Ag^+^ treatment increased intracellular H_2_O_2_, we asked if this contributed to cell death caused by AgNP or Ag^+^ exposure. SUM159 and iMEC cells were co-treated with PEG-catalase and AgNPs or Ag^+^ for 24 h and cell viability was assessed by MTT assay. PEG-catalase treatment did not affect AgNP-mediated cytotoxicity in SUM159 (Fig. 5A) or iMEC cells (Fig. 5B), and there was no significant change in the IC_50_ of AgNP treatment for either cell line (Fig. 5C). However, PEG-catalase decreased the cytotoxicity of Ag^+^ in both SUM159 (Fig. 5D) and iMEC cells (Fig. 5E). The IC_50_ of Ag^+^ exposure to SUM159 cells increased twofold in the presence of PEG-catalase, and there was a threefold increase in IC_50_ for Ag^+^ exposure to iMEC cells in the presence of PEG-catalase (Fig. 5F). Clonogenic growth of SUM159 and iMEC cells after AgNP or Ag^+^ exposure with or without PEG-catalase also was evaluated. In agreement with results from the MTT assay, PEG-catalase did not alter clonogenic growth of both iMEC and SUM159 cells following AgNP exposure (Fig. 5G, H), but did protect both cell lines from Ag^+^ (Fig. 5I, J).

**Fig. 6:**
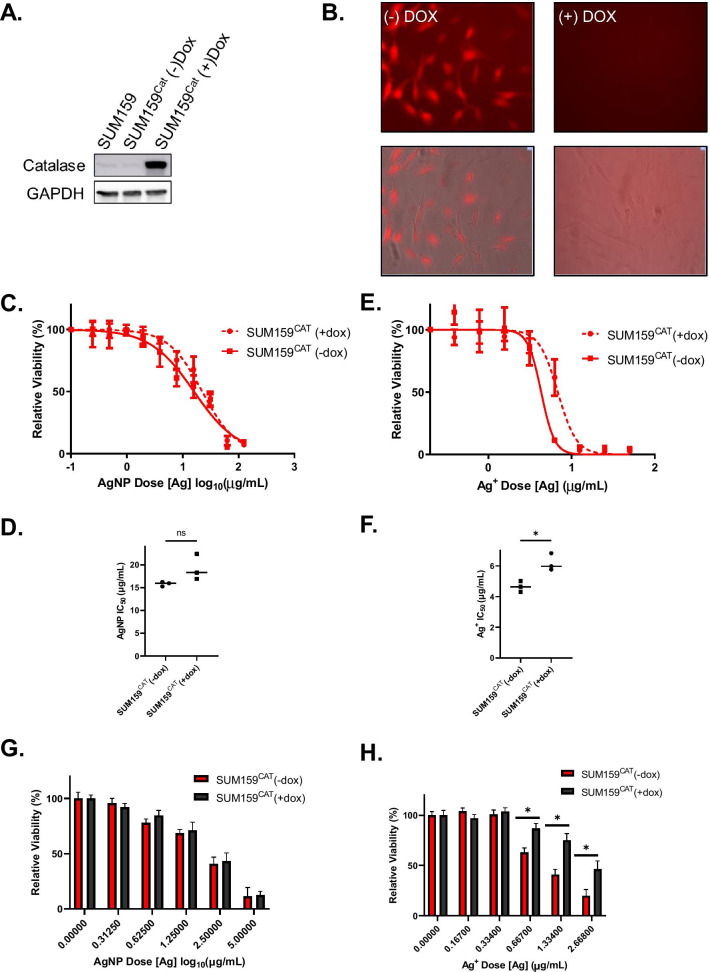
Reduction of hydrogen peroxide by over-expression of catalase catalase attenuates Ag^+^ but not AgNP mediated cell death. **A** Catalase expression was determined by western blot in parental SUM159, SUM159^Cat^ (-)Dox, and SUM159^Cat^ (^+^)Dox cells. **B** SUM159^Cat^ cells cultured with or without doxycycline were incubated with PBS containing PO1 and fluorescence was measured using confocal microscopy. **C**, **D** SUM159^Cat^ cells cultured with or without doxycycline were exposed to increasing concentrations of **C** AgNPs or **E** Ag^+^ for 72 h. Cell viability was quantified by MTT assay. IC_50_’s were calculated for **D** AgNPs or **F** Ag^+^ using GraphPad prism. Data used to calculate IC50s were obtained from 6 technical replicates and 3 independent experiments (biological replicates). Statistical analysis was performed by Students T-test. Statistical differences are indicated (*p < 0.05). **G**, **H** Long-term proliferative potential was assessed via clonogenic assay after 24 h exposure to **G** AgNPs or **H** Ag^+^ in SUM159^Cat^ cells cultured with or without doxycycline. Data are presented as relative surviving fraction based upon clonogenic growth normalized to plating efficiency. Statistical analysis was performed by two-way ANOVA followed by post-hoc Tukey Test. Statistical differences are indicated (*p < 0.05)

We further evaluated the effect of catalase on sensitivity to AgNPs and Ag^+^ using genetically modified SUM159 cells engineered to express doxycycline-inducible catalase (SUM159^cat^). Increased expression of catalase after doxycycline treatment was verified by immunoblotting (Fig. 6A), and reduction of baseline H_2_O_2_ level was verified by PO1 staining (Fig. 6B). Similarly to what was observed with the addition of PEG-catalase, over-expression of catalase did not affect AgNP-mediated cytotoxicity (Fig. 6C) but was protective against Ag^+^ (Fig. 6D). There was no significant change in IC_50_ of AgNP treatment (Fig. 6E), but catalase overexpression increased the IC_50_ of Ag^+^ (Fig. 6F). Clonogenic growth assays confirmed that catalase over-expression did not protect SUM159^cat^ cells from AgNPs (Fig. 6G), but was protective against Ag^+^ (Fig. 6H). Taken together, these data indicate that production of H_2_O_2_ plays a causal role in Ag^+^ mediated cytotoxicity but is not crucial for AgNP-mediated cytotoxicity.

### AgNPs but not Ag^+^ induce lipid peroxidation

**Fig. 7:**
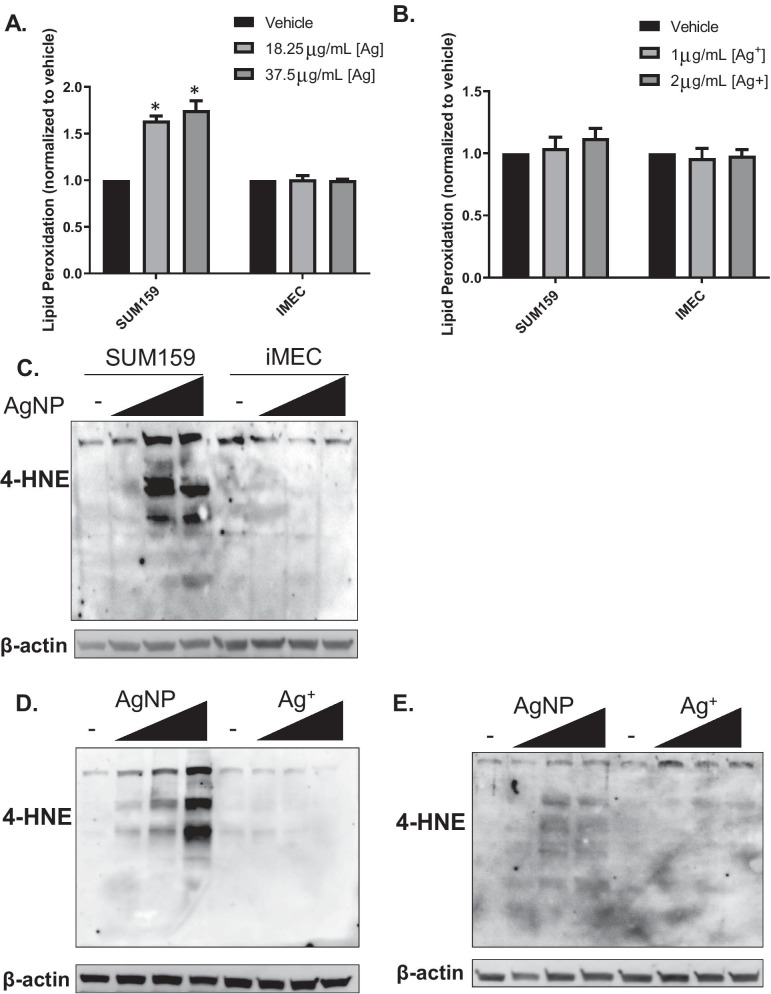
AgNPs, but not Ag^+^, cause lipid peroxidation. To assess lipid peroxidation, SUM159 and iMEC cells were treated with **A** AgNPs or **B** Ag^+^ for 24 h, stained with Liperfluo, and fluorescence was measured using flow cytometry. Statistical analysis was performed by two-way ANOVA followed by post-hoc Tukey Test. Statistical differences are indicated (*p < 0.05). **C** Western blotting to detect 4-HNE was performed on lysates from SUM159 and iMEC cells exposed for 24 h to increasing doses of AgNPs (untreated, 18.25, 37.5 and 75 μg/mL, left to right for each cell line). Western blotting to detect 4-HNE also was performed on **D** SUM159 or **E** iMEC cells exposed to AgNPs (untreated, 18.25, 37.5 and 75 μg/mL, left to right) or Ag^+^ (untreated, 0.5, 1, 2 μg/mL, left to right) for 24 h. β-actin was used as a loading control. Data are representative of a minimum of two experiments

Because H_2_O_2_ did not contribute to the cytotoxicity of AgNPs, we asked if lipid peroxides could play a role. We used Liperfluo, a fluorescent probe that is specific for detecting lipid peroxides [39], to quantify changes in lipid peroxidation following AgNP or Ag^+^ exposure. We observed a significant (*p* < 0.05) increase in lipid peroxidation in AgNP treated SUM159 cells compared to vehicle, but there was no change in lipid peroxides in similarly treated iMEC cells (Fig. 7A), which is consistent with their relative sensitivities to AgNPs. No change in lipid peroxidation was observed in SUM159 and iMEC cells following Ag^+^ treatment (Fig. 7B). 4-Hydroxynonenal (4-HNE) is a toxic endproduct of lipid peroxide decomposition. 4-HNE readily forms protein adducts as both Michael addition products and Schiff bases. We observed a dose dependent increase in histidine adducts of 4-HNE in SUM159 cells exposed to AgNPs, but little change was observed in iMEC cells (Fig. 7C). Consistent with the lack of lipid peroxidation, there was no increase in histidine adducts of 4-HNE following Ag^+^ exposure in either SUM159 (Fig. 7D) or iMEC cells (Fig. 7E). These data show that intact AgNPs cause lipid peroxidation, which correlates with overall sensitivity to AgNP exposure. In contrast, Ag^+^ exposure did not cause lipid peroxidation regardless of relative sensitivity of cell lines to Ag^+^.

### Protein oxidation and aggregation due to AgNPs or Ag^+^ exposure differs between cell lines and correlates with their relative sensitivity to AgNPs or Ag^+^

**Fig. 8:**
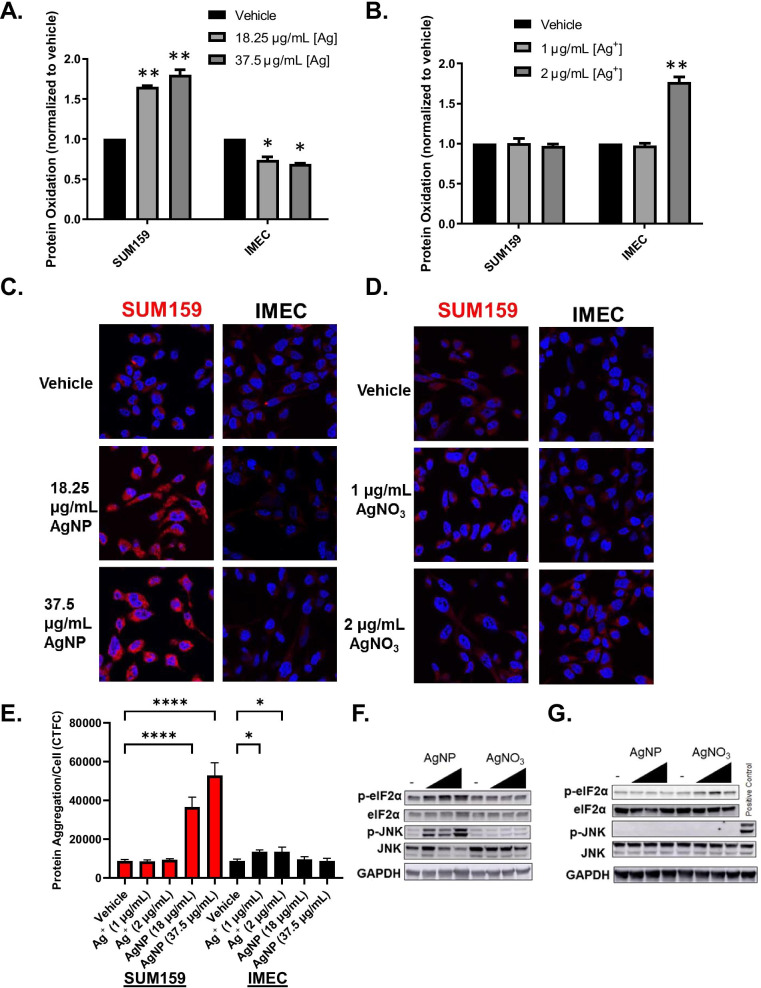
Protein oxidation and aggregation are induced by AgNPs and Ag^+^ at different levels in SUM159 and iMEC cells in proportion to the relative sensitivity of the cells to each toxicant. To assess protein oxidation, SUM159 and iMEC cells were treated with **A** AgNPs or **B** Ag^+^ for 24 h, stained with DCP-NEt2C, and fluorescence was measured using flow cytometry. Data are representative of three independent experiments. Statistical analysis was performed by two-way ANOVA followed by post hoc tukey test. Statistical differences are indicated (*p < 0.05; **p < 0.01). SUM159 and iMEC cells were treated with **C** AgNPs or **D** Ag^+^ for 24 h and protein aggregation was measured by confocal microscopy. **E** Fluorescence images were quantified using ImageJ. Data is plotted as total cell fluorescence ± SEM relative to untreated controls of at least 10 cells per image for 3 images. Statistical analysis was performed by one-way ANOVA followed by post-hoc Sidaks test. Significant differences are indicated (**p < 0.01; *p < 0.05; ****p < 0.0001). Western blotting was used to detect proteotoxic stress responses in **F** SUM159 cells or **G** iMEC cells exposed to AgNPs (untreated, 18.25, 37.5 and 75 μg/mL, left to right) or Ag^+^ (untreated, 0.5, 1, 2 μg/mL, left to right) for 24 h. GAPDH was used as a loading control. Data are representative of a minimum of two independent experiments

Both H_2_O_2_ and lipid peroxides can spread direct effects of AgNPs or Ag^+^ and cause damage including oxidation of thiols in proteins leading to generation of sulfenic acids [40]. Thiol oxidation and formation of 4-HNE adducts can induce protein misfolding and aggregation [41, 42]. Because iMEC and SUM159 cells exhibited opposite relative sensitivities to AgNPs and Ag^+^, we wondered if AgNPs and Ag^+^ would also induce distinct patterns of protein oxidation and aggregation that would correlate with relative sensitivity of the cells to these toxicants. To assess protein oxidation, we used DCP-NEt2-C, a fluorescent probe that is specific for imaging mitochondrial protein sulfenylation [35]. In agreement with their relative sensitivity to AgNPs, there was a significant, dose dependent increase in protein oxidation in SUM159 cells following AgNP exposure (Fig. 8A). For iMEC cells, there was a small decrease in protein oxidation (Fig. 8A). As expected based upon their relative sensitivity, the opposite pattern was observed following exposure of cells to Ag^+^. There was no change in protein oxidation in SUM159 cells exposed to Ag^+^ at either dose, but there was nearly a twofold increase in protein oxidation in iMEC cells exposed to a 2 µg/ml dose of Ag^+^ (Fig. 8B). Similarly, imaging and quantification of protein aggregation using proteostat, a dye that fluoresces after intercalation into hydrophobic pockets formed by misfolded or aggregated proteins, also correlated with differences in sensitivity of cell lines to AgNPs or Ag^+^ (Fig. 8C,D,E). Specifically, there was a dose dependent increase in protein aggregation in SUM159 cells but not in iMEC cells treated with AgNPs (Fig. 8C,E). In contrast, Ag^+^ exposure did not affect protein aggregation in SUM159 cells, but a modest increase was observed in iMEC cells (Fig. 8D, E).

The accumulation of misfolded proteins is cytotoxic and cells will activate stress response programs to mitigate damage. This includes activation of the integrated stress response (ISR), indicated by phosphorylation of eIF2α [43], and mitogen-activated protein kinase (MAPK) signaling pathways, indicated by phosphorylation of c-Jun N-Terminal Kinase (JNK) [44]. As shown in Fig. 8F, AgNP exposure, but not Ag^+^, increased both p-eIF2α and p-JNK in SUM159 cells. In contrast, Ag^+^ exposure, but not AgNPs, increased peIF2α levels in iMEC cells (Fig. 8G). No change in pJNK was observed in iMEC cells after AgNP or Ag^+^ exposure. Protein oxidation and accumulation of protein aggregates following Ag^+^ exposure were modest in iMEC cells compared to what was observed for AgNP treated SUM159 cells, and lack of pJNK may be because damage was below the threshold needed for activation of this response. Overall, both AgNP and Ag^+^ treatment induced protein oxidation, aggregation, and proteotoxic stress responses, but the effects were distinct from one another, dependent on cell type, and were observed to occur in proportion to the relative sensitivity of cell lines to each toxicant.

### AgNPs and Ag^+^ induce distinct forms of cell death

**Fig. 9:**
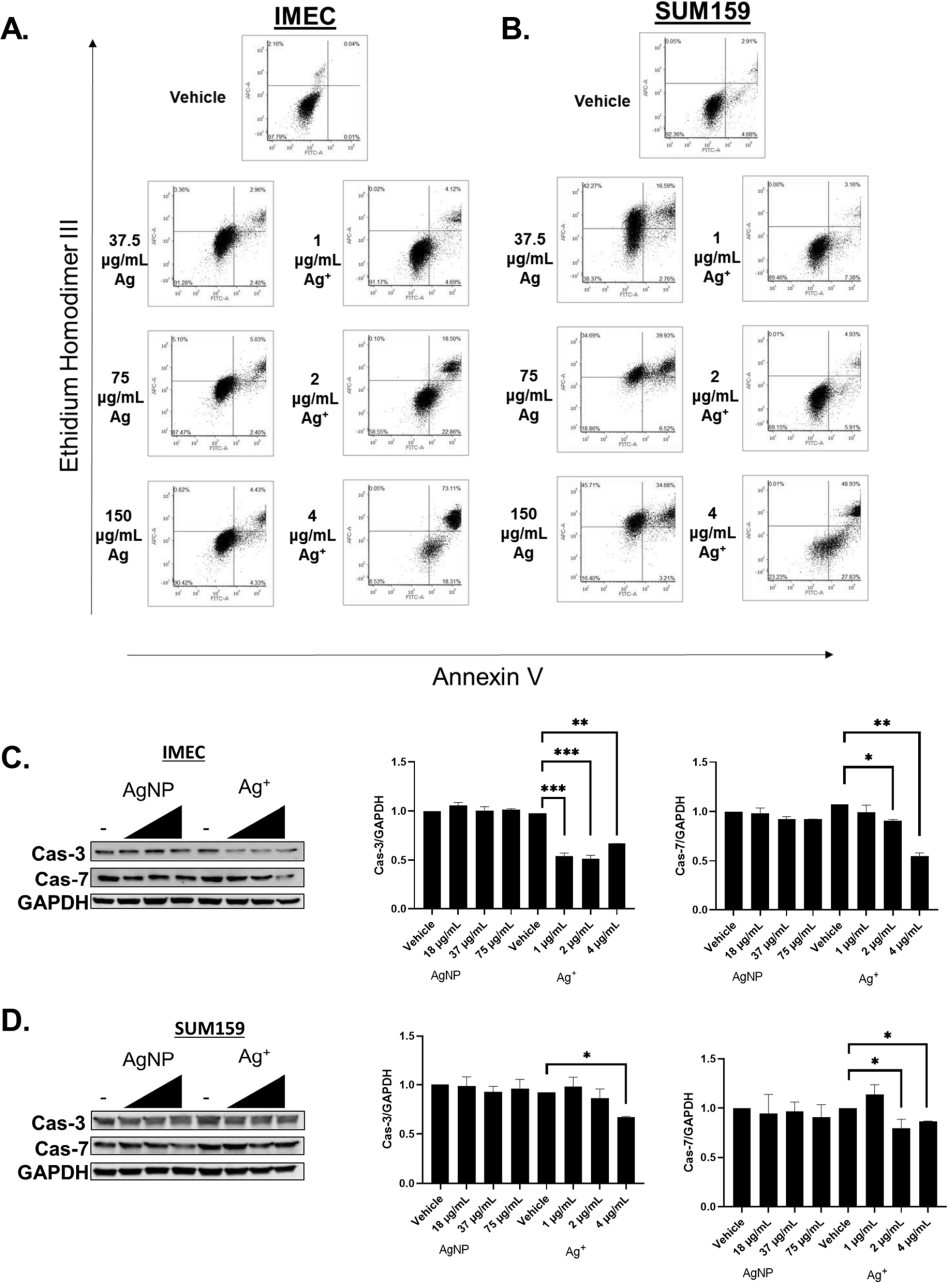
The mechanism of cell death induced by AgNPs differs from that of Ag^+^. **A** iMEC or **B** SUM159 cells were exposed to AgNPs or Ag^+^ for 24 h, co-stained with PI and AnnV, and then evaluated by flow cytometry. The percentages of cells characterized as viable (lower-left quadrant), early apoptotic (lower-right quadrant), late apoptotic (upper-right quadrant), and necrotic (upper left quadrant) are shown within each quadrant. Data are representative of a minimum of two independent experiments. **C** iMEC or **D** SUM159 cells were exposed to AgNPs or Ag^+^ for 24 h and full length caspase expression was detected by western blot. Protein levels relative to GAPDH loading control were quantified by densitometry. Expression of cas-7/GAPDH and cas-3/GAPDH is shown relative to levels detected in untreated control. Statistical analysis was performed using one-way ANOVA followed by post-hoc tukey test. Statistical differences are indicated (*p < 0.05, **p < 0.01, ***p < 0.001)

Because AgNPs and Ag^+^ induced distinct forms of damage to cells, we asked if there were differences in the types of cell death caused by each. AnnexinV (AnnV) and ethidium homodimer III (EthD-III) co-staining was performed on iMEC and SUM159 cells exposed to AgNPs or Ag^+^ for 24 h (Fig. 9A, B). In agreement with the relative insensitivity of iMEC cells to AgNPs, there was little change in early-stage apoptosis (AnnV^+^/ EthD-III^−^ staining, lower right quadrant), primary necrosis (AnnV^−^/ EthD-III^+^ staining, upper left quadrant), or late-stage apoptosis/secondary necrosis (AnnV^+^/ EthD-III^+^ staining, upper right quadrant), even at the highest dose tested. Following Ag^+^ exposure, iMEC cells exhibited dose dependent increase and progression from early-stage apoptosis to late-stage apoptosis/secondary necrosis without an increase in primary necrosis, indicating apoptotic cell death. A different pattern was observed for SUM159 cells after AgNP and Ag^+^ exposure. AgNPs caused a dose dependent increase and progression of both primary necrosis and late-stage apoptosis/secondary necrosis without increasing early-stage apoptosis, indicating necrotic cell death. Although SUM159 cells were less sensitive to Ag^+^ than iMEC cells, similar dose dependent increases and progression from early-stage apoptosis to late-stage apoptosis/secondary necrosis without an increase in primary necrosis supported apoptosis as the mechanism of cell death induced by Ag^+^.

AgNPs may affect the intensity of scattered light detected by flow cytometry, and quantification of changes in side scattered light has been proposed as a metric for determining AgNP uptake [45]. Consistent with previous studies, dose dependent increases in side scatter (but not forward scatter) were detected for both SUM159 and iMEC cells following AgNP exposure (Additional file 1: Fig. S5). Because AgNP treatment only affected AnnV/ EthD-III staining of SUM159 cells but not iMEC cells, changes in side scattering are unlikely to have caused this result. Studies performed by Goering and colleagues at the United States Food and Drug Administration support use of flow cytometry for assessment of AgNP induced apoptosis/necrosis, but caution that that formation of AgNP aggregates could affect data analysis [46]. Ordinarily, no gating based upon forward or side scatter is used for flow cytometry analysis of apoptosis/necrosis. However, gating can be used to eliminate interference due to small particulates caused by AgNP aggregation. We did not detect AgNP aggregates by DLS (Fig. 1), nor were any particulates indicative of AgNP aggregates detected in the forward and side scatter profiles of AgNP-treated SUM159 or iMEC cells (not shown). Therefore, we did not perform any gating prior to AnnV/EthD-III analysis.

To verify the flow cytometry results, we examined pro-apoptotic caspase signaling by western blot in AgNP and Ag^+^ treated cells as a second metric to detect apoptosis. We observed decreased levels of full-length caspases 3 and 7, which is indicative of caspase cleavage, following Ag^+^ exposure, but not after AgNP exposure, in iMEC (Fig. 9C) and SUM159 cells (Fig. 9D). Cleavage of caspases 3 and 7 is a key step for execution of apoptosis. The flow cytometry and western blot data indicated that Ag^+^, but not AgNPs, induced signs of apoptosis in SUM159 cells and iMEC cells. These data show AgNPs and Ag^+^ initiate distinctly different forms of cell death.

## Discussion

The goals of our study were: (i) to determine precisely how AgNPs kill mammalian cells; (ii) to provide definitive proof as to whether or not this mechanism was dependent upon the presence of extracellular Ag^+^; (iii) and to understand how AgNP and Ag^+^ damage propagated through cells. Two critical issues have hampered previous efforts to achieve these goals. The first is contamination of AgNP suspensions with Ag^+^, which prevents separating nanoparticle specific effects from those due to Ag^+^. The second is the fact that some cell lines exhibit greater or lesser sensitivity to AgNPs and Ag^+^ compared to other cell lines, and thus not all cells respond similarly to exposure. To address these issues, we identified AgNPs with negligible Ag^+^ contamination, and evaluated the responses to AgNPs and Ag^+^ in two cell lines that differed in their relative sensitivity to AgNPs and Ag^+^. We found that SUM159 cells, which are a triple-negative breast cancer (TNBC) cell line, were approximately 6.5-fold more sensitive to AgNP exposure compared to iMECs, an immortalized, non-neoplastic breast epithelial cell line. We further found that SUM159 cells were 5.6-fold less sensitive to Ag^+^ compared to iMEC cells, indicating that high sensitivity to AgNPs was not predictive of similar sensitivity to Ag^+^. Exposure to either AgNPs or Ag^+^ increased indications of protein thiol oxidation, accumulation of misfolded proteins, and activation of the integrated stress response in SUM159 or iMEC cells in proportion to the relative sensitivity of the cell lines to each toxicant. However, there were distinct differences between how AgNP and Ag^+^ damage spread throughout the cells. Both Ag^+^ and AgNP exposure increased H_2_O_2_ levels in these two cell lines, but catalase rescued cells from Ag^+^ cytotoxicity and had no effect on the cytotoxicity of AgNPs. This indicates that H_2_O_2_ contributed to the mechanism of action of Ag^+^ but did not play a dominant role in the cytotoxic effects of AgNPs. Instead, our data support a mechanism by which damage from AgNP exposure propagates through cells by generation of lipid peroxides, subsequent lipid peroxide mediated oxidation of proteins, and via generation of 4-HNE protein adducts. 4-HNE can diffuse out of endosomes or lysosomes and into the cytosol, enabling spread of AgNP-induced damage from endosomes to other organelles including mitochondria [42].

In the past, failure to account for Ag^+^ contamination in AgNP dispersions before administration to cells or animals likely contributed to the contradicting data in the AgNP vs Ag^+^ debate. For example, Beer et al. characterized a wide variety of commercially available and laboratory synthesized AgNPs and found that Ag^+^ made up 2.6–5.9% of typical laboratory-made AgNP dispersions, and various commercial preparations AgNP contained 39–69% Ag^+^ by mass [6]. A recent study found that 70% of the commercially available colloidal silver products tested contained exclusively Ag^+^ with no evidence of AgNPs [47]. Because AgNPs used in our study had extremely low amounts of dissolved Ag^+^ (< 0.001% by mass even after storage), we were able to independently examine Ag^+^ and AgNP-dependent mechanistic effects.

An additional confounding factor in assessment of the toxic effects of nanomaterials, including AgNPs, is that they can interfere with assays reliant upon optical absorbance, fluorescence, or luminescence measurements [48–51]. Nanoparticles themselves could absorb or scatter light, fluoresce, or increase autofluorescence in cells. The potential for nanoparticles to affect background absorbance or fluorescence measurements in part can be accounted for by subtracting background measurements taken from label-free, nanoparticle treated cells as we have done in the studies reported here. Nanoparticles also may increase or quench fluorescence from certain fluorophores, quench luminescence, or potentially affect the reaction rates for formation of substrates used to assess cell viability [48–51]. Because these types of interference are challenging to assess under experimental conditions used for cell-based assays, cell-free systems commonly are used. For example, when dispersed in PBS, Mello et al. observed that incubation of AgNPs with MTT caused a dose dependent increase the absorbance of 540 nm wavelengths of light [50]. This is the wavelength most strongly absorbed by the solubilized formazan salt produced by reduction of MTT. Although the authors concluded that certain types of AgNPs could interfere assessment of cell viability based upon spectroscopic quantification MTT reduction, generation of formazan was not chemically verified, nor was any mechanism proposed by which AgNPs could increase the reduction rate of MTT. Therefore, it is unclear if AgNPs directly reduced MTT or if there were other factors which could affect optical absorbance under the experimental conditions tested. In contrast to Mello et al., investigations by Andraos et al. of the interference of AgNPs dispersed in media containing XTT (2,3-Bis-(2-methoxy-4-nitro-5-sulfophenyl)-2H-tetrazolium-5-carboxanilide), which is a tetrazolium salt used in assays similar to the MTT assay, did not identify any change in reduction of XTT due to AgNPs. However, they cautioned that under some conditions, AgNPs could mask the absorbance due to XTT or its formazan reduction product, possibly by adsorption, leading to overestimation of cytotoxicity [49].

Cell-free approaches could be misleading because they often are performed in the absence of biomolecules that are present in the extracellular environment or within cells that dictate the nanoparticle protein corona, formed by adsorption of molecules in the dispersant onto the surface of nanomaterials [52]. For AgNPs, both intra and extracellular organosulfur-containing molecules strongly adsorb or bind to the nanoparticle, altering surface properties that influence colloidal stability (e.g. ζ-potential; hydrophobicity/hydrophilicity) and cell interactions, changing spectroscopic properties, and potentially affecting interactions with dyes or reagents used for performing viability assays [53]. Cell-free approaches also fail to account for confinement of nanoparticles into specific subcellular compartments and assume that all components within the assay system can freely interact. Therefore, assessment of the potential for one type of nanoparticle to interfere with various spectroscopic assays in one specific environment is not inherently generalizable to similar nanoparticles or different environments.

In the absence of an ideal system for identifying all sources of nanoparticle interference with each assay, use of multiple, complimentary metrics to evaluate specific aspects of cell responses to nanoparticles is the best available option. Here, we assessed effects of AgNPs on growth and cell death using optical absorbance (MTT assay for cell growth), fluorescence (AnnV/PI staining as indicators of phosphatidylserine exposure and loss of cell membrane integrity respectively), and combined these with complimentary, non-spectroscopic methods of growth and apoptosis including the label-free clonogenic assay, and quantification of caspase cleavage by western blot. All four assays indicated that AgNPs were significantly more cytotoxic to SUM159 cells than to iMEC cells. Based upon the differences in responses to AgNPs of the two cell lines, it is unlikely that interference of AgNPs with any assay played a dominant role. For example, a dose of 37.5 µg/ml decreased MTT assay absorbance measurements by 70% for SUM159 cells, but no significant reduction in absorbance was observed for identically treated iMEC cells, indicating that effects of AgNPs were cell line dependent. No increase in absorbance, which could potentially be indicative of increased formazan formation, was observed at any of the AgNP doses tested. In a previous study, using AgNPs identical to those used here, we directly compared the results of the MTT assay with both clonogenic growth and label-free, live cell imaging over time for assessment of the cytotoxicity of AgNPs in a panel of four TNBC and two breast epithelial cell lines [54]. All three assays were in agreement regarding the relative sensitivity of the cell lines to AgNPs. In the same study, quantification of the uptake of AgNPs by these cell lines using inductively coupled plasma mass spectroscopy revealed that there was no correlation between the mass of AgNPs taken up and the AgNP IC_50_ across the six cell lines. Interference with assays due to physicochemical properties of the nanomaterial would be expected to correlate with nanoparticle uptake and be independent of cell line/cell type. Thus, our current and previous results provide strong support for a biological basis for observed differences in cytotoxicity rather than effects driven by nanoparticle interference.

In addition to assessing cytotoxic effects of AgNPs, we evaluated AgNP induced proteotoxicity using three complimentary methods. For the first, we used DCP-NEt2C, a probe we developed that is specific for labeling of sulfenic acids in mitochondrial proteins indicative of oxidation. DCP-NEt2C previously was evaluated for detecting AgNP induced protein oxidation and is suited for live cell biological imaging applications because it does not cause mitochondrial toxicity itself, nor is it affected by changes in mitochondrial membrane potential, which could be caused by AgNP exposure [35]. Although DCP-NEt2C is specific for detection of oxidized thiols in mitochondria, we observed DCP-Net2C labeling to be correlated with other metrics of total thiol oxidation in lung cells treated with AgNPs [13]. Furthermore, we previously found that an increase in sulfenylated proteins following AgNP treatment was characteristic of AgNP sensitive breast and lung cancer cells using biotinylated, dimedone-based probes to detect protein sulfenic acids by western blot [9], and by quantification of total reversible protein thiol oxidation using mass spectrometry [14]. The second method we used here to assess proteotoxicity involved staining with proteostat after paraformaldehyde fixation for the formation of protein aggregates in AgNP-treated cells. Proteostat is a type of “turn-on” fluorophore called a molecular rotor. When free to move, fluorescence is low, but when proteostat is entrapped in a hydrophobic pocket of a protein aggregate, motion is restricted, and the molecule becomes highly fluorescent. Increased proteostat fluorescence was detected only in AgNP treated SUM159 cells, and not in AgNP treated iMEC cells or in untreated cells, indicating dependency on both AgNP exposure and cell type. Lastly, we used western blotting to determine if the protein oxidation and protein aggregation observed by flow cytometry or confocal fluorescence microscopy activated cell stress pathways in response to proteotoxicity. All three metrics indicated that AgNPs increased proteotoxicity in SUM159 cells at doses that did not affect iMEC cells. The agreement between the three assays, which were performed in live cells, fixed cells, or in cell lysates, greatly strengthens the accuracy of conclusions drawn regarding AgNP induced proteotoxicity compared to use of any single assay.

To understand the potential cause of protein oxidation, we assessed the effects of AgNPs on total ROS, H_2_O_2_, and lipid peroxides. Detection of ROS presents specific challenges due to need for quantification in live cells. In our studies, we used two different dyes for evaluation of ROS: CM-H_2_DCF-DA and PO1. Oxidation of H_2_DCF yields a highly fluorescent derivative, DCF, which is commonly used to quantify overall ROS, though there are several caveats including: lack of specificity for detection of H_2_O_2_; potential for oxidation by other several one-electron oxidizing species; potential for transition metals or peroxidases to catalyze its oxidation; and DCF radicals can react with oxygen to generate ROS [55]. In contrast, boronate-based fluorescent probes like PO1 directly react with H_2_O_2_, which causes boronate deprotection resulting in a highly fluorescent derivative [56]. Significant increases in DCF and PO1 fluorescence were detected by confocal laser scanning microscopy in both SUM159 and iMEC cells after AgNP or Ag^+^ exposure. We validated the specificity of the dyes for detecting H_2_O_2_ through use PEG-catalase treatment and genetically over-expressed catalase, both of which eliminated treatment-induced increases in fluorescence for each dye. Because there was no evidence that the AgNPs used in our studies directly increased oxidation of either dye in the presence of catalase, the increased fluorescence was likely due to increased ROS rather than an artifact due to nanoparticle interference. Under some, cell-free conditions, AgNPs may quench DCF fluorescence [49], and therefore our results may underestimate ROS or H_2_O_2_ production, though this would not affect our overall conclusions.

We used flow cytometry to detect oxidation of phospholipids in cell membranes using a lipid peroxide-specific fluorescent probe called Liperfluo. Following quantification of changes in Liperfluo fluorescence by flow cytometry, we detected a significant increase in fluorescence indicative of lipid peroxidation in AgNP treated SUM159 cells but not in iMEC cells. As a non-fluorescent means of validating our data, we quantified 4-HNE adducts by western blot, and again observed an increase in 4-HNE adducts in SUM159 cells, but not in iMEC cells. Ag^+^ exposure did not increase Liperfluo fluorescence or 4-HNE adducts in either cell line, despite increasing both H_2_O_2_ and total ROS levels, providing additional evidence that these observations were indicative of lipid peroxidation and not merely a reflection of changes in the overall redox state of the cells following AgNP exposure.

Some studies suggest that ROS generated after AgNP exposure is a critical component of their cytotoxic mechanism [15, 22, 25]. In contrast, we show that both Ag^+^ and AgNPs increase H_2_O_2_, but only Ag^+^-induced cell death was rescued by catalase. Previous studies using glutathione (GSH) or N-acetyl cysteine (NAC) as antioxidants to mitigate damage due to AgNP-induced ROS may be misleading because both can directly chelate free Ag^+^ or bind to AgNPs [28]. These effects, rather than ROS mitigation, may explain previous observations that GSH or NAC reduce AgNP cytotoxicity. NAC also increases GSH levels, and GSH plays a critical role in detoxifying lipid peroxides [32] as well as 4-HNE adducts [57], and thus mitigating effects on AgNP-induced lipid peroxides also may contribute to their capacity to rescue AgNP-induced cell death. In well-controlled studies comparing the potential genotoxicity of PVP-coated AgNPs to Ag^+^, Li et al. also concluded that the mechanisms by which AgNPs and Ag^+^ induced genotoxicity differ from each other [27]. They showed that there was minimal release of Ag^+^ from their AgNPs, and that AgNPs themselves, but not Ag^+^, were capable of increasing hydroxyl radicals in cells, further supporting a nanoparticle specific mechanism of action. Importantly, they observed that Trolox, a vitamin E analogue, protected against both AgNP and Ag^+^ toxicity, but NAC protected only against Ag^+^. Vitamin E and its analogues act as peroxyl radical scavengers capable of terminating chain reactions caused by lipid peroxyl radicals abstracting a hydrogen atom from a neighboring polyunsaturated fatty acid in a lipid membrane. Thus, the protective effects of Trolox against AgNPs observed by Li et al. [27] are consistent with a lipid peroxide driven mechanism of AgNP toxicity. However, vitamin E is unable to block the initial generation of lipid peroxyl radicals, nor does it prevent degradation of lipid peroxides into toxic end products such as 4-HNE. Furthermore, vitamin E is not specifically an antioxidant for lipid peroxides but also is involved in multiple biological processes, and in some cases acts as a pro-oxidant as previously reviewed [58]. Because AgNPs, but not Ag^+^, cause increases in lipid peroxidation and 4-HNE-adduct formation, it is clear that different forms of damage are induced. To fully establish causality, it will be necessary to perform additional studies to modulate the lipid profile of AgNP sensitive or insensitive cells in order to enrich or deplete lipid membranes for highly oxidizable lipid species.

The AgNPs used for the majority of our studies consist of only two components: a nominal 25 nm silver core and a dense stabilizing layer of PVP, a polymer considered generally safe by the United States Food and Drug Administration, making these nanoparticles suitable for further biomedical development. For these studies, PVP serves the purpose of stabilizing the AgNPs such that when the dry powder is dispersed in aqueous solution, the nanoparticles remain well dispersed (unaggregated) under physiologically relevant ionic strength and pH, and do not behave as a larger sized aggregate rather than as nanoscale particles. One challenge in generalizing findings regarding the toxicity profile of nanomaterials is that the specific physicochemical and toxicological properties of a nanoparticle depend not only on the material making up the core, but also on the size, shape, coating, presence of contaminants, and the environment in which testing is performed. However, in an earlier study [11], we found that the TNBC-selective cytotoxic property of AgNPs was retained by particles with various sizes (5–150 nm diameters), shapes (spheres and triangular prisms), or capping agents (PVP, chitosan, and silica). The environment in which AgNPs are dispersed also may affect their dissolution, and alter their toxicity profile. For example, inhaled AgNPs may dissolve, release Ag^+^, and potentially reform into smaller nanoparticles due to interaction with biomolecules in the lung extracellular fluid [59, 60]. Precisely how AgNP toxicity differs when assessed in varying physiological environments remains to be determined.

We and others previously showed that AgNPs were highly cytotoxic to multiple TNBC cell lines at doses that were not toxic to non-neoplastic breast cells [9–11, 54]. Thus, an extension of our current work on the mechanism of AgNP-induced cell death is the potential to exploit this knowledge for breast cancer therapy. In earlier studies demonstrating the sensitivity to TNBC cells to AgNPs [10, 11], it also was observed that AgNPs induced endoplasmic reticulum (ER) stress, which occurs when misfolded proteins accumulate in the ER. Because AgNPs do not localize to the ER, it was unclear how ER stress was induced. Misfolded proteins can accumulate in the ER if protein degradation machinery, including the ubiquitin proteasome system and autophagy, is overwhelmed by damaged proteins generated elsewhere in the cell [61]. We observed that AgNPs increased lipid peroxides in TNBC SUM159 cells, but not in iMEC cells. These lipid peroxides can oxidize proteins, or degrade to 4-HNE, which reacts with proteins to form adducts. Both types of damage cause protein misfolding and aggregation and can trigger ER stress and ISR through eIF2α signaling. The results of our cell death studies show that AgNPs induce necrosis in SUM159 cells at doses that were not toxic to iMEC cells. This is consistent with several studies that show that AgNPs induce necrosis [62, 63]. In contrast, Ag^+^ treatment caused apoptosis at lower doses in iMEC cells compared to SUM159 cells. Accumulation of 4-HNE and the resulting buildup of protein aggregates in the cell are highly toxic and have been shown to initiate necrotic cell death [64, 65], whereas cell death caused by excess ROS is more often mediated by apoptosis [66]. Therefore, the difference in cell death pathways could be due to the induction of lipid peroxidation by AgNPs but not Ag^+^. TNBCs are enriched in long-chain, polyunsaturated fatty acids (PUFAs) [67], which are prone to peroxidation, and this may be an underlying factor that drives their sensitivity to AgNPs. Accumulation of lipid peroxides is also involved in a form of non-apoptotic, iron-dependent, programmed cell death called ferroptosis [68, 69]. It is not known whether AgNPs initiate ferroptosis and additional studies also will be necessary in order to confirm this.

Although we show mechanistic differences between responses of cells exposed to AgNPs or extracellular Ag^+^, we are unable to rule out effects caused by Ag^+^ released from AgNPs after they are taken up. Additionally, the precise reason why exposure to AgNPs but not Ag^+^ causes lipid peroxidation remains to be identified. This may be driven by the different uptake pathways of AgNPs and Ag^+^, which would result in localization to distinct sites in the cells. AgNPs are taken up by phagocytic and endocytic pathways [18] and reside in membrane bound vesicles. AgNPs may directly oxidize unsaturated fatty acids in endosomal membranes or degrade in endosomes to release Ag^+^ in high concentration, which then reacts with unsaturated fatty acids. In contrast, Ag^+^ can enter cells through copper ion transporters [16, 17] and accumulate in the cytosol. When Ag^+^ is taken up as an ion, lack of proximity or low concentration of Ag^+^ near endosomal lipid membranes may limit effects of Ag^+^ on lipid peroxidation, or Ag^+^ may rapidly react with thiols in the cytosol rather than with lipids. The tools necessary to measure intracellular silver ions and silver ion-ligand species are now emerging [20, 70, 71], but direct measurement of intracellular dissolution of AgNPs remains a major challenge [8].

## Conclusion

In conclusion, our integrated approach to assessing AgNP and Ag^+^ cytotoxicity indicates distinct differences exist in the responses of mammalian cells to AgNPs and Ag^+^. Specifically, AgNPs drive cell death through a mechanism that involves lipid peroxidation, proteotoxic stress, and necrotic cell death, whereas Ag^+^ exposure increases H_2_O_2,_ which drives oxidative stress and apoptotic cell death. This work identifies a specific mechanism by which AgNPs kill mammalian cells that is not dependent upon the contribution of Ag^+^ released in extracellular media. Understanding precisely which factors drive the toxicity of AgNPs is essential for biomedical applications such as cancer therapy, and of importance to identifying consequences of unintended exposures.

## Supplementary Information

**Additional file 1.** Supplementary Figures S1-S5.

## Data Availability

Data sharing is not applicable to this article as no datasets were generated or analysed during the current study.
