# Supplementary material for: The mechanism of cell death induced by silver nanoparticles is distinct from silver cations
Source: Part Fibre Toxicol. 2021 Oct 14;18:37. doi: 10.1186/s12989-021-00430-1 (PMC8515661; doi:10.1186/s12989-021-00430-1)
Supplement: Supplementary file 1 — Additional file 1. Supplementary Figures S1-S5. [file 12989_2021_430_MOESM1_ESM.pdf]

Supplementary Figure S1

A.

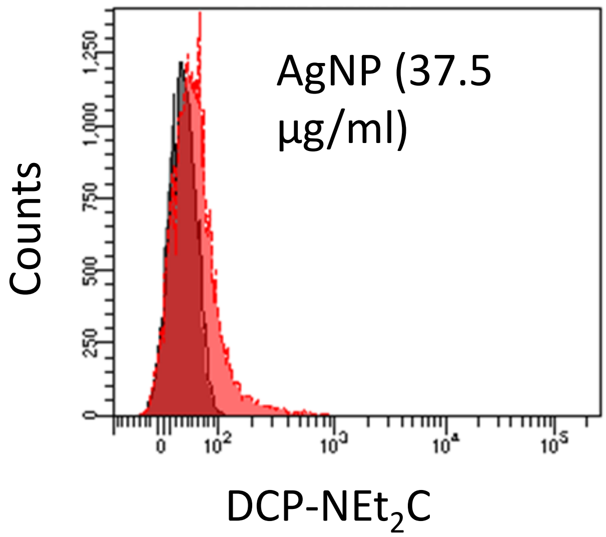

B.

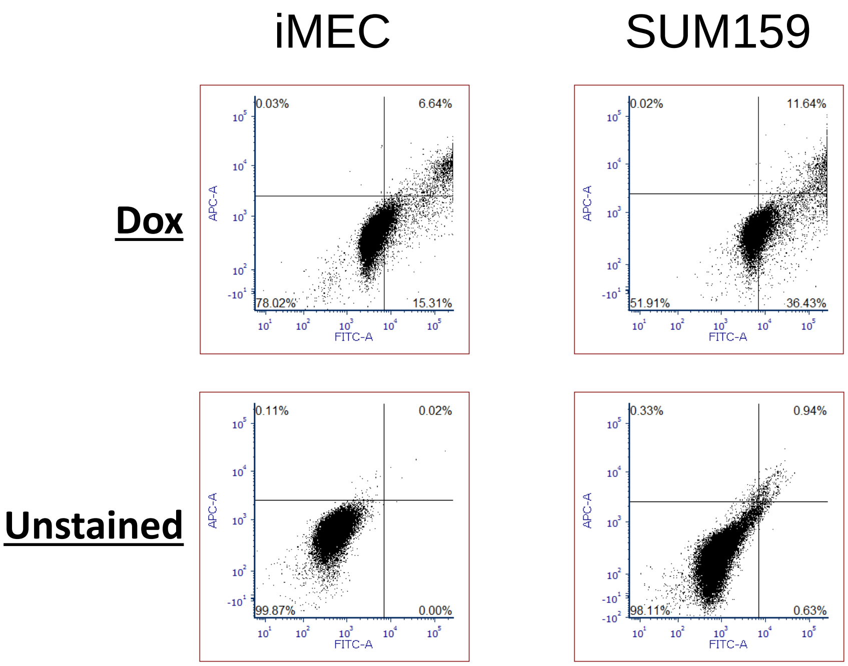

Supplemental Figure S1. *Controls demonstrating lack of interference of AgNPs with flow cytometry measurements.* (A) Background fluorescence of untreated or AgNP treated (37.5 µg/ml) was quantified under conditions identical to those in figure 8A,B but without staining with DCP-NEt_2C . Mean fluorescence levels of unstained cells were less than 0.5% of mean fluorescence levels of DCP-NEt_2C stained controls, and 0.25% of DCP-NEt_2C stained, AgNP-treated cells. This indicates that background fluorescence from AgNPs did not contribute to the increase in fluoresce in DCP-NEt_2C stained, AgNP treated cells. (B) *Positive and unstained controls for Annexin V/PI.* SUM159 and iMEC cells were exposed to doxorubicin (1 µg/mL) or AgNPs (150 µg/mL) for 24 h, costained with PI and AnnV, and then evaluated by flow cytometry. The percentages of cells characterized as viable (lower-left quadrant), early apoptotic (lower-right quadrant), late apoptotic (upper-right quadrant), and necrotic (upper left quadrant) are shown within each quadrant. These data show that staining is specific for detection of apoptosis, and that AgNPs do not affect the fluorescence of the AnnV of PI stains. Data are representative of a minimum of two independent experiments.

Supplementary Figure S2

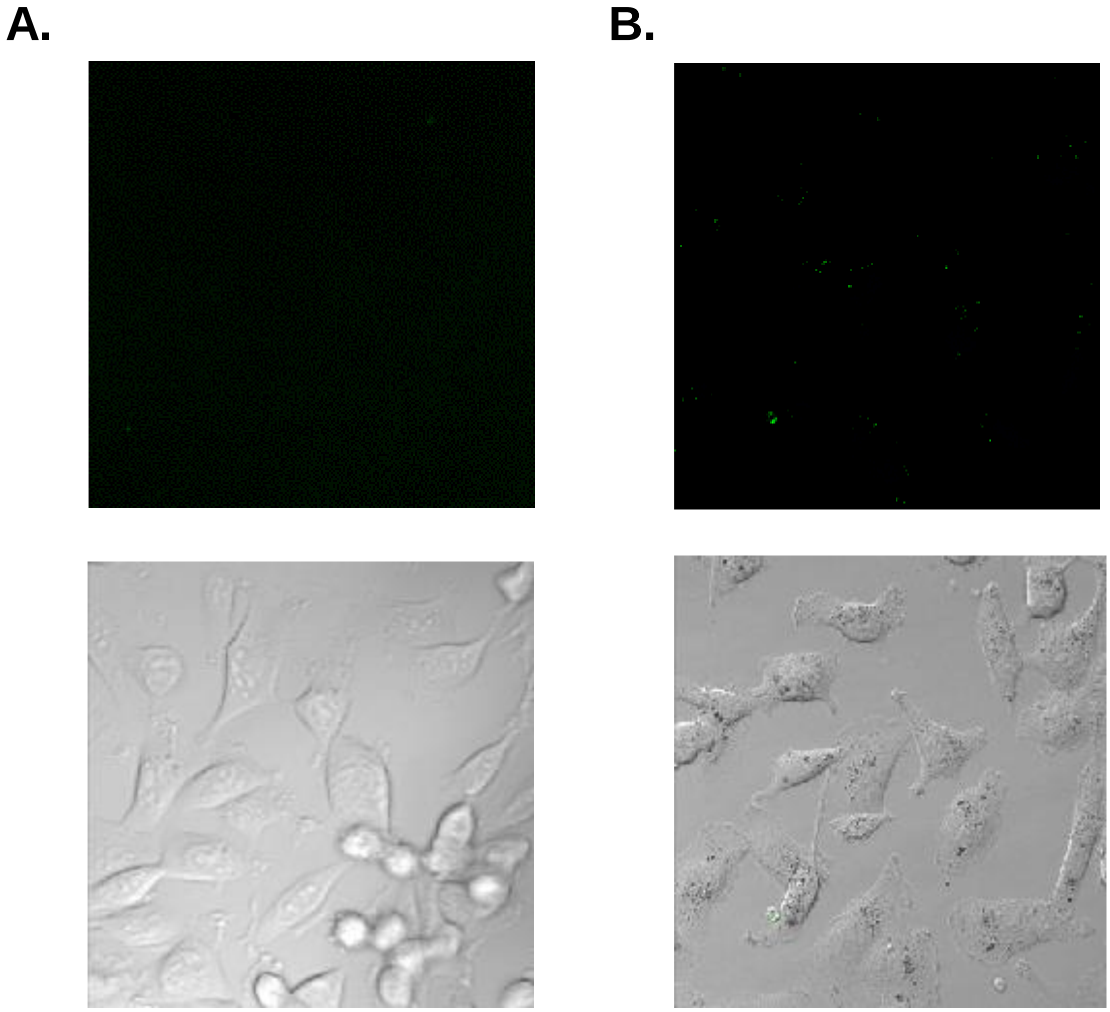

Supplemental Figure S2. *AgNPs exhibit no autofluoresence.* **(A)** SUM159 and **(B)** iMEC cells were treated with AgNPs for 24 h. Fluorescense (RFP/GFP) was measured using confocal microscopy.

Supplementary Figure S3

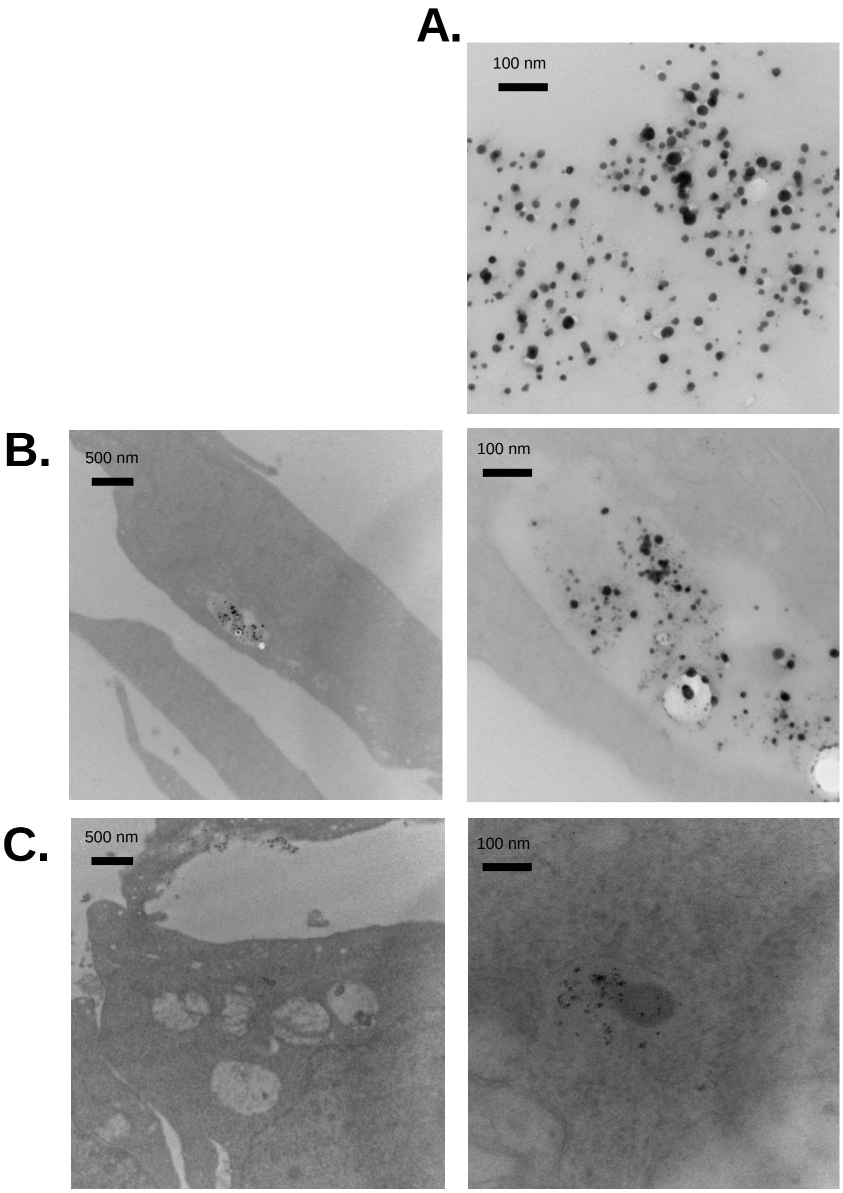

Supplemental Figure S3. *Imaging of uptake and trafficking of AgNPs in SUM159 and iMEC cells*. **(A)** Electron micrographs show 25nm AgNPs outside of the cell. **(B)** Electron micrographs show AgNPs in endolysosomal vesicales in iMEC cells after 1 h pulse and 5 h chase at 4800 X magnification or 30000 X magnification. **(C)** Electron micrographs show AgNPs in endolysosomal vesicles in SUM159 cells after 1 h pulse and 5 h chase at 4800 X magnification or 30000 X magnification.

Supplementary Figure S4

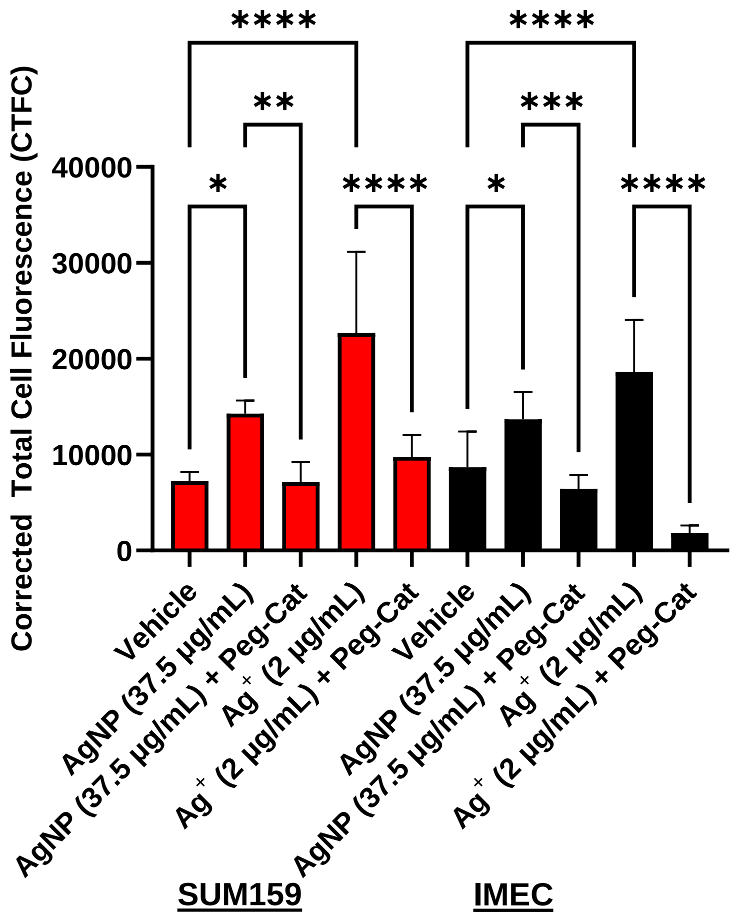

Supplemental Figure S4. *Production of hydrogen peroxide following AgNP or Ag+ treatment.* SUM159 and iMEC cells were treated with either Ag^{+} or AgNPs for 24 h with and without 100 IU peg-catalase. Cells were then incubated with PBS containing PO1 for 30 min and fluorescence was measured using confocal microscopy. Data are displayed as corrected total cell fluorescence (CTFC) and is representative of triplicate independent experiments.

Supplementary Figure S5

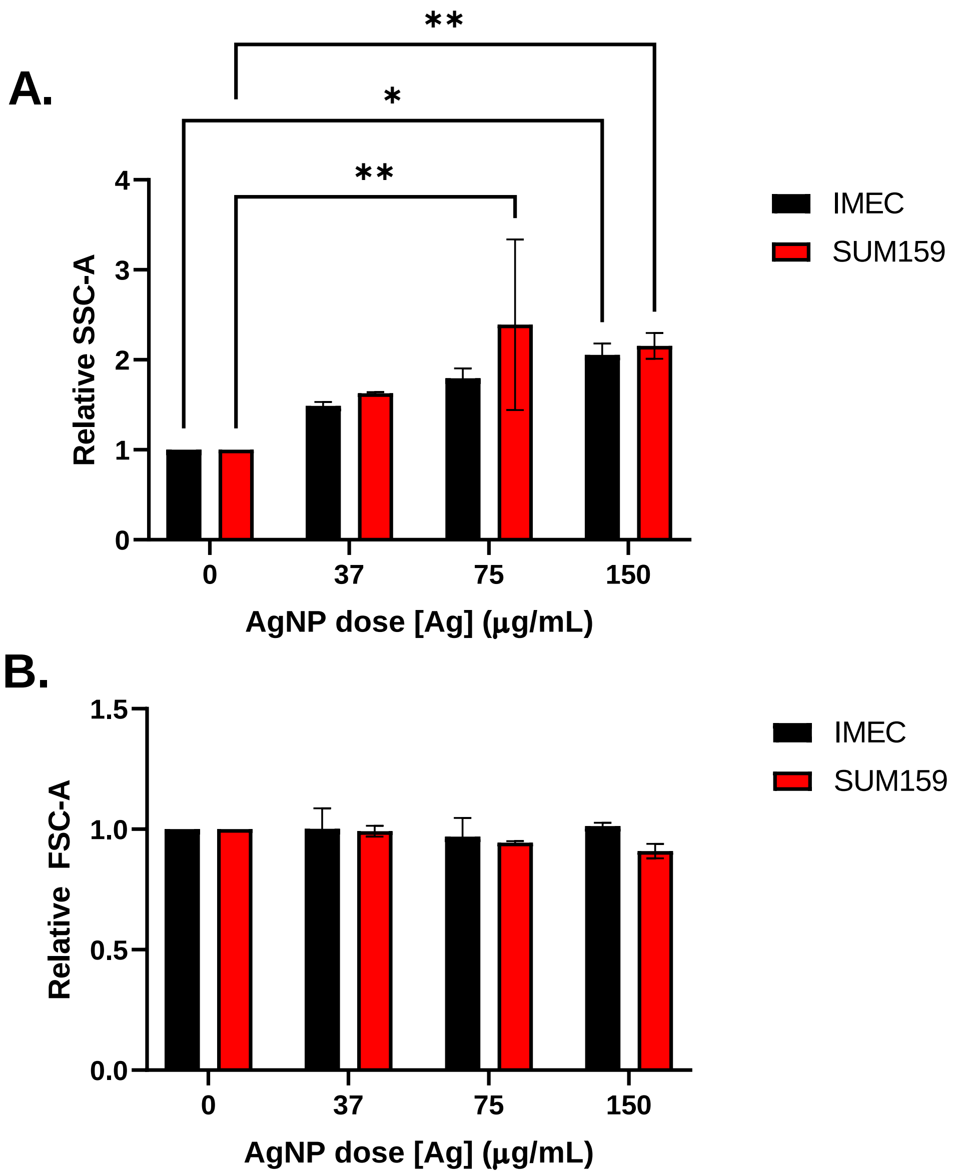

Supplementary Figure S5. *Effect of AgNP uptake on flow cytometry-based side and forward scatter plots*. SUM159 and iMEC cells were treated with AgNPs for 24 h, washed extensively, and **(A)** side scatterering (SSC-A) or **(B)** forward scattering of light was quantified by flow cytometry. Data are displayed as the relative mean intensity of scatterered light in comparison to the untreated control. Four independent replicates were used. Statistical analysis was performed by two-way ANOVA followed by post-hoc Tukey test. Significan differences (* p<0.05; ** p<0.01) are indicated.
